# Identification of ferroptosis and drug resistance related hub genes to predict the prognosis in Hepatocellular Carcinoma

**DOI:** 10.1038/s41598-023-35796-z

**Published:** 2023-05-29

**Authors:** Chengjun Li, Xiaomeng Cui, Yarui Li, Dan Guo, Shuixiang He

**Affiliations:** Department of Gastroenterology, the First Affiliated Hospital of Xi’an Jiaotong University, Xi’an, 710061 Shaanxi China

**Keywords:** Cancer, Computational biology and bioinformatics, Genetics

## Abstract

Hepatocellular carcinoma (HCC) is the third leading cause of cancer-related death worldwide. Currently, overcoming the drug resistance in HCC is a critical challenge and ferroptosis has emerged as a promising therapeutic option for cancer. We aim to construct a new gene signature related to ferroptosis and drug resistance to predict the prognosis in HCC. The RNA-seq data of HCC patients was obtained from the Cancer Genome Atlas database. Using least absolute shrinkage and selection operator cox regression, Kaplan–Meier analysis, and differential analysis, we constructed a prognostic model consisting of six hub genes (TOP2A, BIRC5, VEGFA, HIF1A, FTH1, ACSL3) related to ferroptosis and drug resistance in HCC. Functional enrichment, pathway enrichment and GSEA analysis were performed to investigate the potential molecular mechanism, and construction of PPI, mRNA-miRNA, mRNA-RBP, mRNA-TF and mRNA-drugs interaction networks to predict its interaction with different molecules. Clinical prognostic characteristics were revealed by univariate, multivariate cox regression analysis and nomogram. We also analyzed the relationship between the signature, immune checkpoints, and drug sensitivity. The expression of the gene signature was detected in HCC cell lines and HPA database. Our prognostic model classified patients into high and low-risk groups based on the risk scores and found the expression level of the genes was higher in the high-risk group than the low-risk group, demonstrating that high expression of the hub genes was associated with poor prognosis in HCC. ROC analysis revealed its high diagnostic efficacy in both HCC and normal tissues. The proportional hazards model and calibration analysis confirmed that the model’s prediction was most accurate for 1- and 3-years survival. QRT-PCR showed the high expression level of the gene signature in HCC. Our study built a novel gene signature with good potential to predict the prognosis of HCC, which may provide new therapeutic targets and molecular mechanism for HCC diagnosis and treatment.

## Introduction

Liver cancer is the most common malignant liver disease and ranks among the top four malignancy types worldwide^1^. Hepatocellular carcinoma (HCC) is the most common type of liver cancer and accounts for about 90% of cases^2^. HCC has an extremely high malignancy rate and is the third leading cause of cancer-related death worldwide^3^. Currently, surgical resection and liver transplantation can treat hepatocellular carcinoma at an early stage. In particular, radiofrequency ablation, hepatic artery chemoembolization, tumor immunotherapy, and molecular targeted therapy offer new opportunities for the treatment of HCC. Although these therapies have significantly improved the clinical outcomes of HCC patients, the overall survival and prognosis of patients are still poor due to the high relapse and metastasis rate. Furthermore, more than 50% of HCC patients have been in the intermediate or advanced stages when diagnosed, with 70% of patients relapsing within the first 5-years of initial treatment^4^. In such cases, many HCC patients miss the opportunities for optimal treatment. Therefore, there is an urgent to continuously search for prognostic biomarkers involved in HCC to provide better therapeutic strategies.

Currently, more than 90% of cancer deaths are caused by chemotherapy failure due to resistance to conventional treatment, and various potential mechanisms for drug resistance include tumor heterogeneity, cellular level changes, genetic factors, growth factors, enhanced DNA repair capacity, enhanced drug efflux, and increased metabolism of xenobiotics^5^. In addition, the tumor microenvironment (TME), proteins and their extensive matrix have been implicated the factors in the development of drug resistance^6^. Each of these can lead to a decrease in the drug treatment effect, thus making tumor treatment more difficult. Although small molecules, peptides, and nanotheranostics as well as targeted delivery systems have emerged to minimize drug resistance in cancer and improve treatment outcomes, there are still limitations to these therapeutic approaches for advanced HCC. In recent years, sorafenib has been considered the first-line treatment for HCC^7^, which is the only FDA-approved drug in advanced HCC, however, it is hindered by the occurrence of drug resistance. Studies have shown that only about 30% of patients benefited from sorafenib, and they usually developed resistance after 3 months of treatment^8^. Over the past few decades, the approved drugs had also failed to inhibit tumor growth due to the emergence of acquired resistance, resulting in high recurrence rates in advanced HCC patients. Therefore, exploring the mechanisms of drug resistance and finding novel molecular targets are urgently needed to improve the prognosis of HCC patients.

In recent years, ferroptosis has attracted widespread attention as a newly discovered form of cell death. It is characterized by an iron-dependent accumulation of intracellular lipid reactive oxygen species (ROS)^9^. As a unique mode of regulatory cell death, distinct from apoptosis, necrosis, and autophagy, ferroptosis is considered to be the most promising tumor inhibitor that can affect tumorigenesis by regulating intracellular iron level and ROS^10^. It has shown great potential in cancer treatment, especially in malignancies that were less sensitive to conventional chemotherapy^11^. Compared with non-resistant cancer cells, those with invasive drug-resistant cancer cells were more likely to be killed by ferroptosis inducers^12^. Moreover, regulating ferroptosis can even reverse resistance to chemotherapy, targeted therapy and immunotherapy^13^. A previous study found that blocking the NRF2/GPX4 pathway promoted ferroptosis to enhance the sensitivity of HCC cells to sorafenib^14^. Another study indicated that silencing metallothionein-1G (MT-1G) enhanced the sensitivity of HCC cells to sorafenib by triggering ferroptosis^15^. In addition, dihydroartemisinin enhanced the inhibitory effect of sorafenib on HCC cells by inducing ferroptosis^16^. Thus, ferroptosis would be a promising strategy to increase the sensitivity of HCC cells to chemotherapy. On the other hand, most of the previous risk predicting models of HCC involved ferroptosis-related genes and non-coding RNAs prognostic signature with immune-related phenotype in hepatocellular carcinoma, but only a few did the drug-resistant analysis^17,18^. For example, Li et al. found a ferroptosis-related long non-coding RNA prognostic signature correlates with genomic heterogeneity, immunosuppressive phenotype, and drug sensitivity, which revealed the crucial role of lncRNAs related to ferroptosis in HCC^19^. Also, Wang et al.^20^ conducted the prognostic characteristics, immune-related, and drug-resistant analysis of ferroptosis-related genes in HCC. Nevertheless, there was no independent model to comprehensively evaluate the gene signature of ferroptosis and drug-resistant phenotypes with HCC prognosis and pathological factors. At the same time, there was no related molecular correlation analysis. Hence, it is necessary to clarify the key role of such genes in HCC to comprehensively understand their molecular mechanisms in the development of tumors.

In the current study, we established an innovative prognostic signature based on ferroptosis and drug resistance in HCC and validated its impact on the risk features and clinical prognosis of HCC patients. Additionally, we conducted a comprehensive analysis of the differential expression, biological function, and molecular interactions of the gene signature to deeply investigate the key mechanisms and signaling pathways. These gene expression was further validated through the GEO, TCGA-LIHC, LIHC-US datasets, and HPA database, as well as molecular biology experiments. Our results will offer novel insights into tumorigenesis and development involved in HCC and will aid in the selection of effective therapies for HCC patients based on novel biomarkers.

## Methods

### Data selection

The gene expression profile for HCC patients were downloaded from the Gene Expression Omnibus (GEO, http://www.ncbi.nlm.nih.gov/geo/) database, which were GSE6222, GSE46408 and GSE65372 datasets. The three datasets (GSE6222, GSE46408 and GSE65372) were respectively derived from homo sapiens mRNA data GPL570 [HG-U133A_2] Affymetrix Human Genome U133A 2.0 Array, GPL4133 Agilent-014850 Whole Human Genome Microarray 4 × 44 K G4112F (Feature Number version), and GPL14951 Illumina HumanHT-12 WG-DASL V4.0 R2 expression beadchip.The GSE6222 dataset included a total of 13 samples, we selected 12 samples for subsequent analysis, which had 10 HCC samples and 2 control normal samples. The GSE46408 dataset included a total of 12 samples, which had 6 HCC samples and 6 control normal samples. The GSE65372 dataset included a total of 54 samples, which had 39 HCC samples and 15 control normal samples.

Besides, we acquired liver hepatocellular carcinoma (LIHC) count sequencing data from the cancer genome atlas (TCGA, https://portal.gdc.cancer.gov/) through the TCGA biolinks package^21^, the data was normalized in FPKM (Fragments per kilo base per million) format. The TCGA-LIHC dataset included a total of 426 samples, including 374 LIHC samples (Group: LIHC) and 50 control samples (Group: Normal). The samples without prognostic data were excluded from the UCSC Xena database (http://genome.ucsc.edu), and the clinical information of 368 samples was obtained.

We used the key word “drug-resistant” to search in the GeneCards (https://www.genecards.org/) database and obtained 1941 drug resistance-related genes. These genes were intersected with those 1978 drug resistance-related genes acquired from the Gene Set Enrichment Analysis (GSEA, http://www.gsea-msigdb.org/) database also using the term “drug-resistant” as a search term. As a result, 14 drug resistance-related genes (UFM1, FANCI, HMGB1, DHFR, TOP2A, BIRC5, GSTP1, VEGFA, HIF1A, PIK3CA, CXCR4, KIT, MMP2, TUBB) were acquired. Then we obtained 619 ferroptosis-related genes from the GeneCards database using the “Ferroptosis” as the search term, and intersected these genes with the 65 ferroptosis-related genes obtained from the GSEA database also using “Ferroptosis” as the search term, then intersected with the 60 ferroptosis-related genes from literature^22^.As a result, we obtained 21 ferroptosis-related genes (AIFM2, GPX4, SLC7A11, TP53, ACSL4, TFRC, NCOA4, HMOX1, ALOX15, IREB2, HSPB1, FTH1, CBS, ACSL3, SAT1, CISD1, AKR1C3, AKR1C2, STEAP3, AKR1C1, GCLC), then we combined the drug resistance-related genes with the ferroptosis-related genes to obtain 35 genes related to ferroptosis and drug resistance (Supplementary Data Sheet S1).

The count sequencing data of liver hepatocellular carcinoma dataset (LIHC-US) was downloaded from the International Cancer Genome Consortium (ICGC) database (https://dcc.icgc.org/), which was normalized to the TPM (Transcripts Per Kilobase of exon model per Million mapped reads) format. The LIHC-US dataset included an expression matrix of 294 LIHC samples and corresponding clinical prognostic information (OS. time).

### Construction of the prognostic model

We selected the overall survival (OS) and gene expression profile data related to ferroptosis and drug resistance from 368 HCC samples with clinical data in the TCGA-LIHC dataset, using ten-fold-cross validation to select seeds 2021 to perform LASSO regression and visualize the results, and the prognosis-related ferroptosis and drug-resistant genes were obtained. The Kaplan–Meier curve (KM curve) for prognosis-related genes were plotted, and we picked the genes with *P* value < 0.05 in the KM curve as the prognosis-related ferroptosis and drug-resistant genes, and the survival time of patients was analyzed and inferred based on the expression of relevant prognostic genes.

### Screening for differentially expressed prognosis-related genes

We used the limma package^23^ to standardize the gene expression profile data of 368 HCC patients in the TCGA-LIHC dataset, and plotted PCA figure to demonstrate the effect before and after standardization. Based on the hazard scores from the LASSO regression analysis, samples with hazard scores higher than the median were classified as high hazard groups (group: high) and samples with hazard scores lower than the median were classified as low hazard groups (group: low). Differential analysis of high and low hazard groups was applied to obtain differentially expressed genes (DEGs) with |logFC|> 0 and *p* value < 0.05. We intersected these DEGs with genes related to ferroptosis and drug resistance to obtain the differentially expressed prognosis-related genes, and then extracted the expression profile data of them in the dataset TCGA-LIHC using the ComplexHeatmap package to plot heatmaps.

### Functional enrichment (GO) and pathway enrichment (KEGG) analysis

GO and KEGG enrichment analysis of differentially expressed prognosis-related ferroptosis and drug-resistant genes was carried out by using the R package clusterProfiler^24^, with the entry screening criteria of *p* value < 0.05 and FDR value (q value) < 0.05, which had statistical significance, and *p* value was corrected by Benjamini- Hochberg (BH).

### Receiver operating characteristic (ROC) curve

The receiver operating characteristic curve (ROC) is a comprehensive indicator of sensitivity and specificity for continuous variables, and the interrelationship between sensitivity and specificity is reflected through mapping. The Area Under Curve (AUC) values generally ranged from 0.5 to 1, and the closer to 1, the better diagnosis.

The time dependent ROC is a special application of ROC curve in the model of survival data. We used the time ROC package to analyze the relationship between the screened prognosis-related genes and the occurrence of HCC, with the ggplot2 package to visualize it.

### Gene set enrichment analysis (GSEA)

We extracted the expression profile data of 368 HCC patients in the TCGA-LIHC dataset for LASSO regression analysis. Then we acquired the c2.cp.v7.2.symbols.gmt gene set from the Molecular Signatures Database (MSigDB) as the reference, and used the clusterProfiler package to make enrichment analysis of the genes grouped by high and low hazard in TCGA-LIHC dataset. The parameters applied in the GSEA were as follows: seeds of 2020, number of calculations of 10,000, minimum number of genes per gene set of 10 and maximum number of genes of 500, and p-value correction of Benjamini-Hochberg (BH). The screening criteria for significant enrichment were *p* value < 0.05 and FDR value (q value) < 0.05. The Top4 results were visualized according to the NES (normalized enrichment score).

### Construction of PPI, mRNA-miRNA, mRNA-RBP, mRNA-TF and mRNA-drug interaction networks

The STRING database was used to construct PPI network of differentially expressed prognosis-related genes (minimum required interaction score: medium confidence (0.400) and the PPI network model was visualized by Cytoscape (version 3.9.1)^25^.

We used the multiMiR package^26^ to search for the obtained prognosis-related ferroptosis and drug-resistant genes in two experimentally validated miRNA-target gene interaction databases (miRTarBase^27^ and TarBase^28^) to acquire miRNAs interacted with the gene signature. Then the intersection of two databases was taken and the mRNA-miRNA interaction network was mapped by Cytoscape software.

ENCORI database was used to predict RNA binding proteins (RBPs) that interacted with the gene signature (TOP2A, BIRC5, VEGFA, HIF1A, FTH1, ACSL3). We visualized the mRNA-RBP interaction network by Cytoscape software with the clusterNum ≥ 5 and clipExpNum ≥ 5.

We searched for transcription factors (TF) that combined with the genes using the ChIPBase (version 3.0) and hTFtarget database. The interaction data with the six genes was downloaded from both databases and the mRNA-TF interaction network was visualized by Cytoscape software.

The direct and indirect drug targets of the gene signature were predicted by CTD (Comparative Toxicogenomics Database) database. Cytoscape software was applied to visualize the mRNA-drugs regulatory network and complete the network construction.

### Clinical correlation analysis

To investigate the clinical prognostic value of differentially expressed genes related to ferroptosis and drug resistance in HCC, we used univariate cox regression analysis of these genes and selected factors with *p* < 0.05 for inclusion in the multivariate cox regression analysis to construct a multivariate cox regression model. Based on the results of the univariate cox regression analysis, we constructed a forest plot. The nomogram was plotted based on the multivariate cox regression analysis to predict the 1-year, 3-years, and 5-years survival of HCC patients. We used the survival package to evaluate the effect of nomogram models on patients’ survival.

We performed the differential analysis and further assessed the impact of the gene expression level on patient prognosis and clinicopathological characteristics. The differential expression of the prognosis-related genes with different clinical features was compared. In particular, we analyzed the impact of the gene expression level on different clinical variables in HCC tissues.

To increase the persuasion of multivariate cox regression results, we utilized the expression matrix of the LIHC-US dataset and corresponding prognostic survival information. Using both univariate and multivariate cox regression analysis to evaluate the expression level of the gene signature, and their correlation with clinical outcomes. The nomogram analysis was plotted to evaluate the univariate and multivariate cox regression results in the LIHC-US dataset. Moreover, we constructed a proportional hazards model and performed calibration analysis for the clinical prognosis at 1, 3, and 5-years.

### Validation of the differentially expressed prognosis-related genes

We integrated the GSE6222 and GSE46408 datasets and employed the limma package to perform normalization on the combined dataset (GSE Combine). The sva package was applied to remove any potential batch effects in the GSE Combine. Subsequently, the normalization was conducted on the GSE65372 dataset.

The grouped comparative graph of the 426 samples from the GSE Combine, GSE65372 and TCGA-LIHC was plotted using LIHC samples (group: LIHC) and control samples (group: Normal) as subgroups to further validate the expression of prognostic-related genes from the TCGA-LIHC dataset.

### Immune check-point and drug sensitivity analysis of the differentially expressed prognosis-related genes

Forty-four immune checkpoints (Supplementary Data Sheet S2) were acquired from the related literature^29^. The gene expression profile data of immune checkpoints from the dataset TCGA-LIHC was extracted. We calculated to get the relevant matrices of immune checkpoint genes and differentially expressed prognosis-related ferroptosis and drug-resistant genes. Correlation heatmaps were plotted to show the results. The correlation scatter plots were drawn for genes with high correlation.

The Gene Set Cancer Analysis (GSCA, http://bioinfo.life.hust.edu.cn/GSCA/) was used to obtain the correlation between the differentially expressed prognosis-related genes and drug IC50 from Genomics of Drug Sensitivity in Cancer database (GDSC. https://www.cancerrxgene.org/), then we plotted bubble plots to visualize the results.

### Cell culture

The human HCC cells including Hep3B, HepG2, Huh7, HCC-LM3, as well as immortalized human liver cells THEL2 were all obtained from American Type Culture Collection (ATCC). Hep3B, HepG2, Huh7 and HCC-LM3 cells were incubated in DMEM high glucose medium with 10% fresh fetal bovine serum. THEL2 cells were incubated in BEGM complete medium. All the cells were cultured in the moist cell incubator at 37 °C with 5% of CO2 for proper time to get the total RNA.

### RNA isolation and quantitative real-time PCR (qRT-PCR)

Trizol reagent (Ambion) was used as the protocol shown to extract total RNAs of cells (Hep3B, HepG2, Huh7, HCC-LM3 and THEL2). Then, inverse transcription was conducted as the procedure of HiScript^®^ II Q Select RT SuperMix for qPCR (VAZYME, China) and the system was established as the protocol of 2 × Q3 SYBR qPCR Master Mix (TOLOBIO, China). In the process β-actin was regarded as internal reference to assess the relative expression of genes involved according to the formula 2^−ΔΔCt^. The sequences of primers used in this study were listed in supplementary Table S1.

### Statistical analysis

Data processing and statistical analysis were performed by R software (version 4.2.1). The statistical significance of normally distributed variables was estimated using independent Student t-tests for comparisons between two groups of continuous variables, and Mann–Whitney u-tests (Wilcoxon rank sum test) were used for differences between non-normally distributed variables. Statistical significance between the two groups of categorical variables was analyzed by using the chi-square test or Fisher's exact test for comparison. The survival package of R was used to perform survival analysis, and the log-rank test was applied to evaluate the significance of the differences between the two groups in survival time. Both univariate and multivariate cox analyses were based on the survival R package and LASSO analyses were based on the glmnet R package^30^. All *P* values were two-sided tests and the differences had statistical significance at *P* < 0.05.

## Results 

### The technical flowchart of the research (Fig. 1)

**Figure 1:**
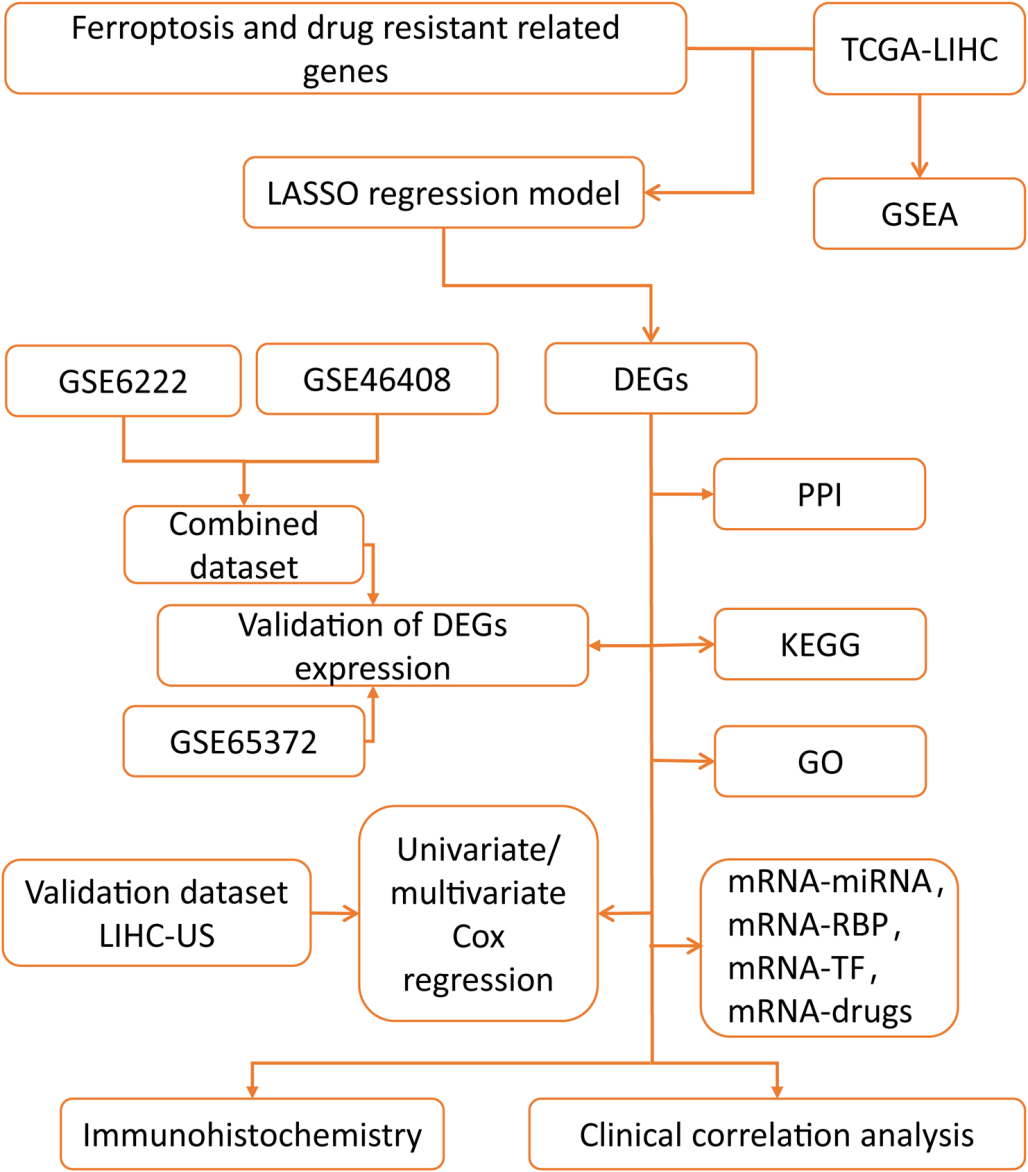
The technical flowchart of the research. LIHC: liver hepatocellular carcinoma; GSEA: Gene Set Enrichment Analysis; LASSO regression model: Least Absolute Shrinkage and Selection Operator regression model; DEGs: differentially expressed genes; PPI: Protein–protein interaction; KEGG: Kyoto Encyclopedia of Genes and Genomes; GO: Gene Ontology; RBP: RNA binding protein; TF: Transcription factors.

In this study, the LIHC samples with comprehensive clinical data were included for further analysis. The flowchart of this study was displayed in Figure 1.

### Construction of the prognostic model for ferroptosis and drug-resistant genes

**Figure 2:**
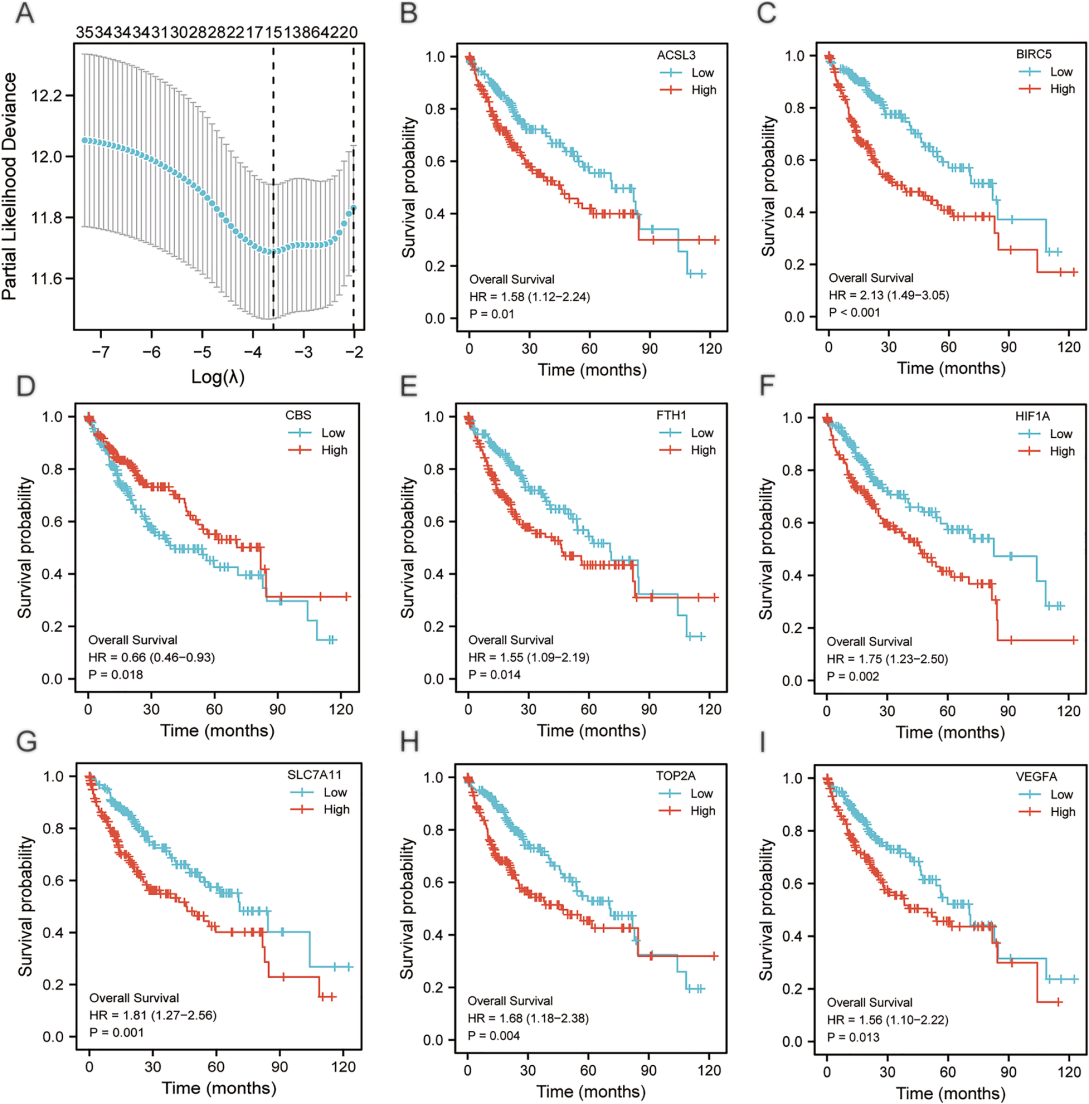
Visualization of LASSO regression results and KM curves. (**A**)The visualization of LASSO regression results in the TCGA-LIHC dataset. (**B-I**) KM curves of the genes (ACSL3, BIRC5, CBS, FTH1, HIF1A, SLC7A11, TOP2A, VEGFA) in TCGA-LIHC dataset, *p* < 0.05; KM: Kaplan–Meier.

 We performed LASSO regression analysis and visualized the results (Fig. 2A).The results showed that a total of 15 genes (TOP2A, BIRC5, VEGFA, HIF1A, KIT, GPX4, SLC7A11, TP53, HMOX1, ALOX15, FTH1, CBS, ACSL3, SAT1, CISD1) were included in the LASSO regression prognostic model, and we plotted Kaplan–Meier curves of the 8 genes among these 15 genes that had statistical significance (*P* < 0.05) based on the prognostic information of dataset TCGA- LIHC. We selected these 8 following prognosis-related ferroptosis and drug-resistant genes ACSL3 (*P* = 0.01, Fig. 2B), BIRC5 (*P* < 0.001, Fig. 2C), CBS (*P* = 0.018, Fig. 2D), FTH1 (*P* = 0.014, Fig. 2E), HIF1A (*P* = 0.002, Fig. 2F), SLC7A11 (*P* = 0.001, Fig. 2G), TOP2A (*P* = 0.004, Fig. 2H), VEGFA (*P* = 0.013, Fig. 2I) that were all with prognostic value.

**Figure 3:**
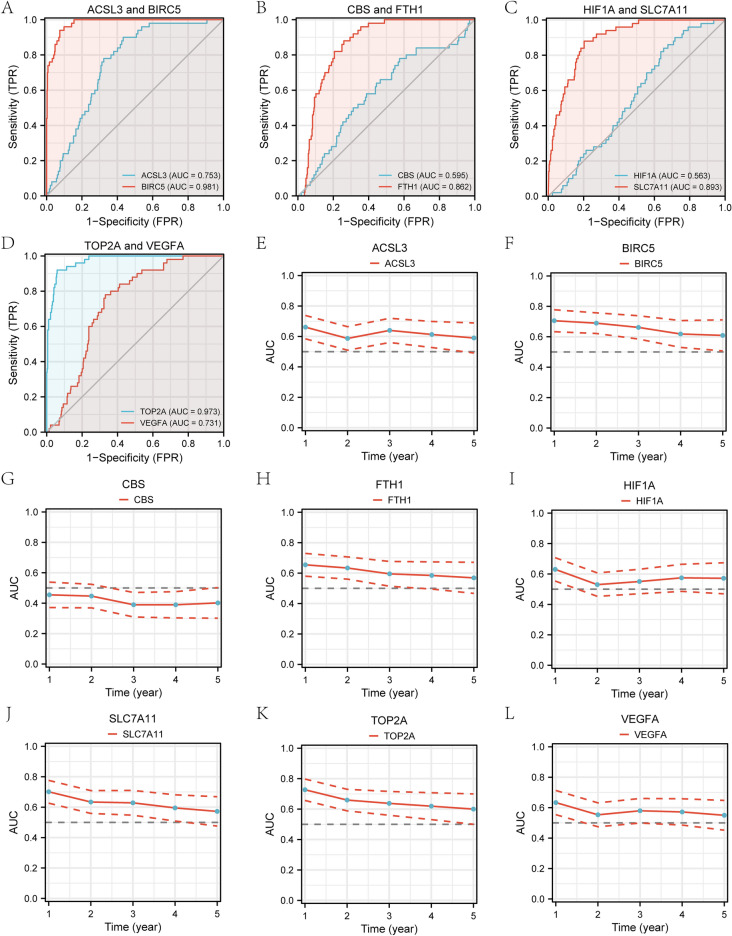
ROC curves and time-dependent ROC line charts for prognosis-related ferroptosis and drug-resistant genes. (**A–D**)Prognostic ROC curves for genes ACSL3, BIRC5, CBS, FTH1, HIF1A, SLC7A11, TOP2A, VEGFA. (**E–L**) Time-dependent ROC line charts for genes ACSL3, BIRC5, CBS, FTH1, HIF1A, SLC7A11, TOP2A, VEGFA. ROC: receiver operating characteristic curve; AUC: Area Under Curve.

The ROC curves for the 8 genes in the TCGA-LIHC dataset displayed that the genes TOP2A (AUC = 0.973, Fig. 3D) and BIRC5 (AUC = 0.981, Fig. 3A) had high accuracy for the diagnosis of HCC. The genes VEGFA (AUC = 0.731, Fig. 3D), FTH1 (AUC = 0.862, Fig. 3B), ACSL3 (AUC = 0.753, Fig. 3A) and SLC7A11 (AUC = 0.893. Figure 3C) were accurate for the diagnosis of HCC. The genes HIF1A (AUC = 0.563, Fig. 3C) and CBS (AUC = 0.595, Fig. 3B) had lower accuracy for the diagnosis of HCC. Time-dependent ROC line charts (Fig. 3E–L) showed the curve area values of the gene expression level varies over time. The gene CBS had a certain inhibitory effect on the occurrence of HCC (Fig. 3G), while the other seven genes (TOP2A, BIRC5, VEGFA, HIF1A, FTH1, ACSL3, SLC7A11) had a certain promoting effect on the occurrence of HCC (Fig. 3E–L).

### Differential expression analysis of prognosis-related ferroptosis and drug-resistant genes

**Figure 4:**
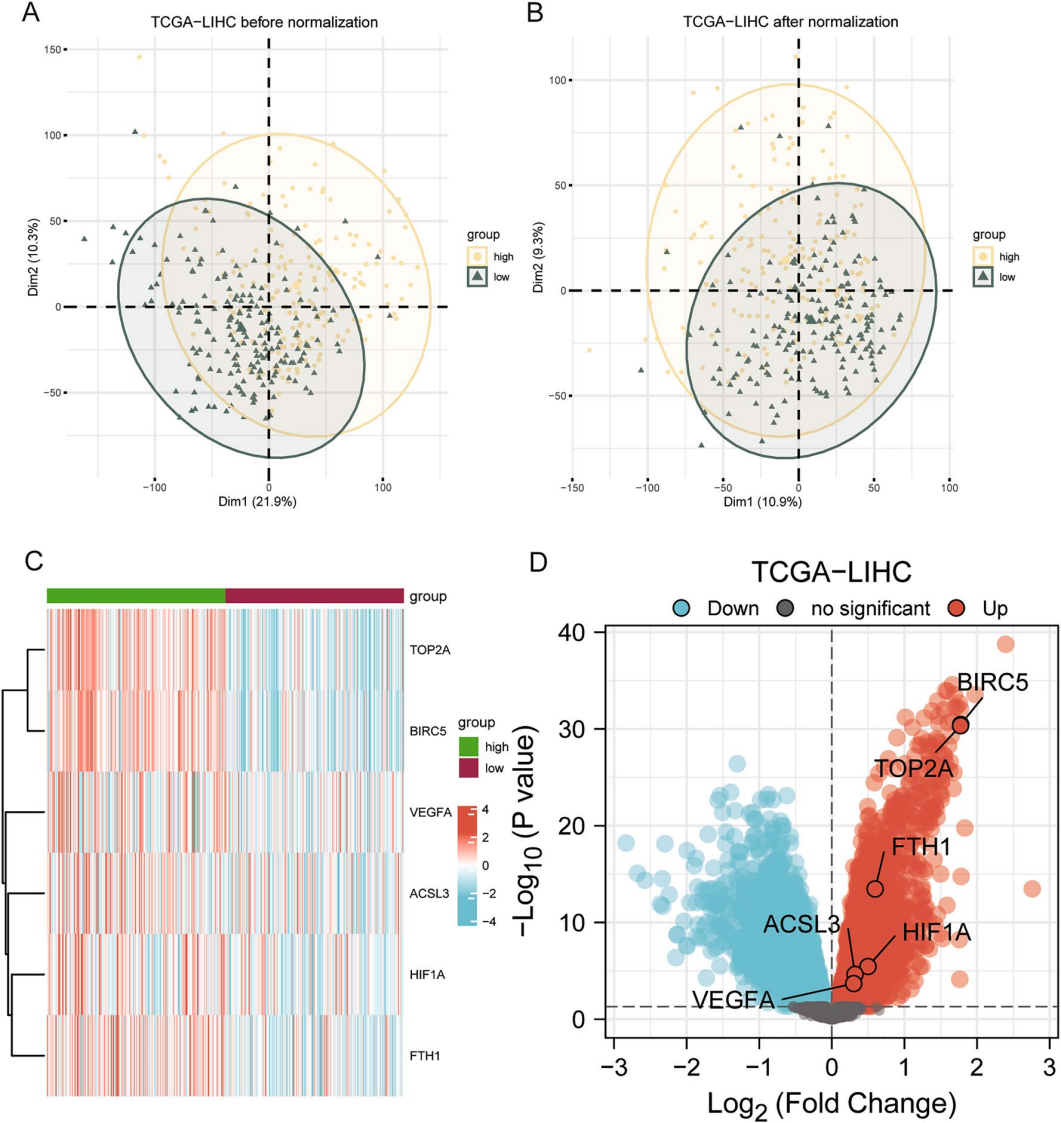
PCA plots and differential analysis heatmaps. (**A**) PCA plot before normalization of dataset TCGA–LIHC. (**B**) PCA plot after normalization of dataset TCGA-LIHC. (**C**) Correlation heatmap of differentially expressed prognosis-related ferroptosis and drug-resistant genes in dataset TCGA-LIHC. (**D**) Volcano map from differential analysis of dataset TCGA-LIHC. Up: up-regulated genes; Down: down-regulated genes.

The PCA plot showed the effect before and after standardization of the gene expression profile data (Fig. 4A–B). The results revealed that the differences between samples in the dataset TCGA-LIHC were significantly reduced before and after normalization. DEGs were obtained by differential analysis with high and low hazard groups (group: high and group: low) and the results were plotted by a volcano map (Fig. 4D). The dataset TCGA-LIHC had a total of 7247 DEGs meeting the criteria |logFC|> 0 and *p* value < 0.05. Under this threshold, there were 3815 up-regulated genes (logFC > 0, *p* < 0.05) and 3432 down-regulated genes (logFC < 0, *p* < 0.05). The DEGs were intersected with prognosis-related ferroptosis and drug-resistant genes to obtain six genes (TOP2A, BIRC5, VEGFA, HIF1A, FTH1, ACSL3). The heatmap (Fig. 4C) showed the differential expression of these six prognosis-related ferroptosis and drug-resistant genes in the dataset TCGA-LIHC.

### Gene function enrichment (GO) and pathway enrichment (KEGG) analysis of differentially expressed prognosis-related genes

**Figure 5:**
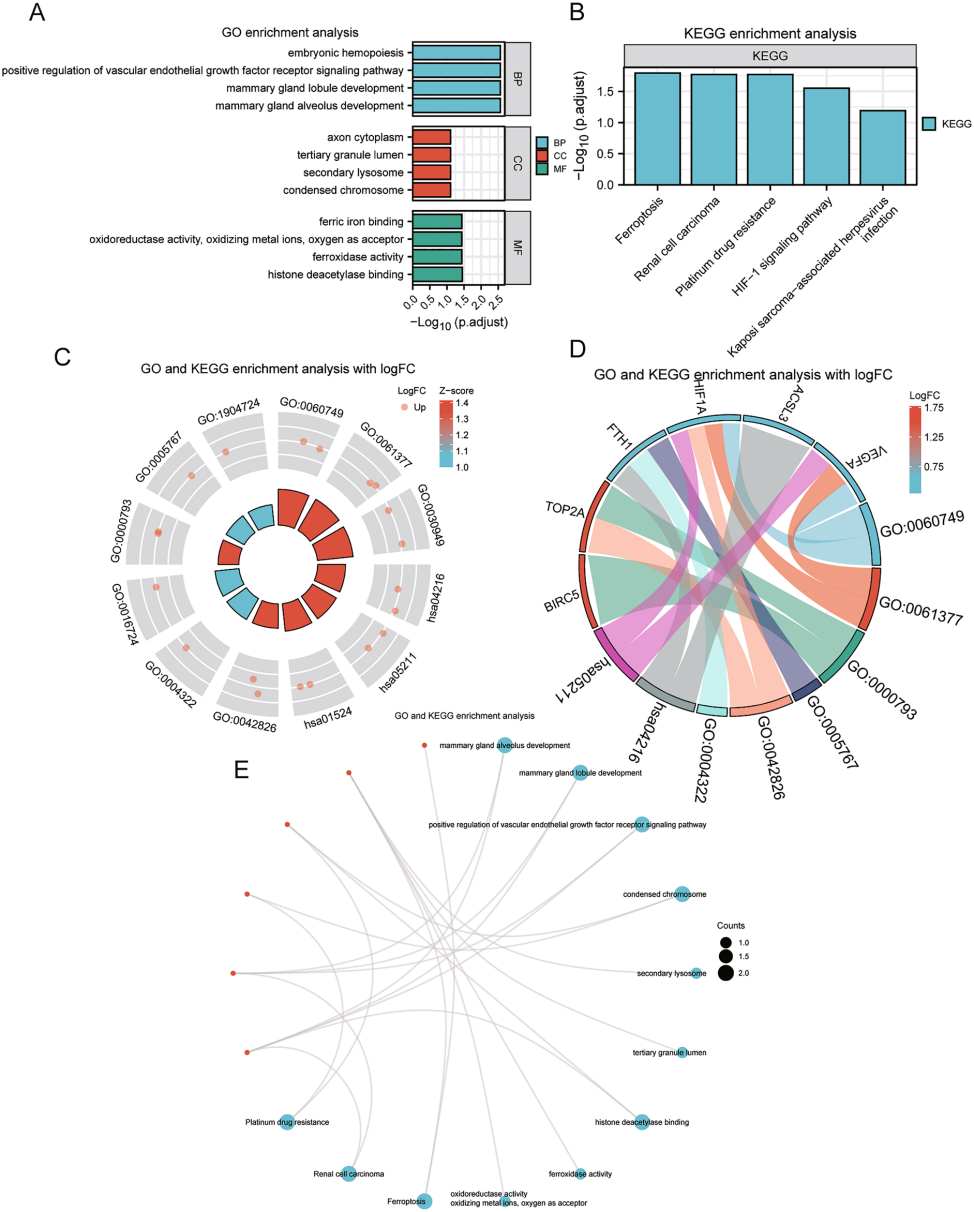
GO and KEGG analysis of differentially expressed prognosis-associated ferroptosis and drug-resistant genes. (**A**) Bar chart of GO analysis for the genes. (**B**) Bar chart of pathway enrichment (KEGG) analysis results for the genes. (**C**) Circle plot of the GO analysis and KEGG analysis combined with logFC results for the genes. (**D**) Chord plot of GO analysis and KEGG analysis combined with logFC results for the genes. (**E**) Ring network of GO analysis and KEGG analysis results for the genes. GO: Gene Ontology; BP: biological process; CC: cellular component; MF: molecular function; Up: up-regulated genes; Down: down-regulated genes.

GO and KEGG enrichment analysis showed that the six genes in HCC were mainly enriched in biological process such as embryonic hemopoiesis, positive regulation of vascular endothelial growth factor receptor signaling pathway, mammary gland lobule development, and mammary gland alveolus development; cellular component such as axon cytoplasm, tertiary granule lumen, secondary lysosome, and condensed chromosome; molecular function such as ferric iron binding, oxidoreductase activity, oxidizing metal ions, oxygen as acceptor, ferroxidase activity, histone deacetylase binding. It was also enriched in Ferroptosis, Renal cell carcinoma, Platinum drug resistance, HIF-1 signaling pathway, Kaposi sarcoma-associated herpesvirus infection pathway. The results of GO enrichment analysis (Fig. 5A) and KEGG enrichment analysis (Fig. 5B.) were visualized by bar chart. (Specific enrichment results were shown in Supplementary Table S2 and GO_KEGG enrichment analysis results).

The network diagrams (Fig. 5E) were drawn based on the results of GO enrichment and KEGG pathway enrichment analysis. The connecting lines illustrated the corresponding molecules and the annotations of the corresponding entries, the larger the node, the more numbers of molecules contained in the entry. Finally, we performed differential analysis to obtain the logFC values of these six genes in the dataset TCGA-LIHC, and combined with logFC of GO analysis to calculate the corresponding standard score (Z-score) for each entry and visualized them by circle plot (Fig. 5C) and chord diagram (Fig. 5D).

### GSEA analysis of the LIHC dataset

**Figure 6:**
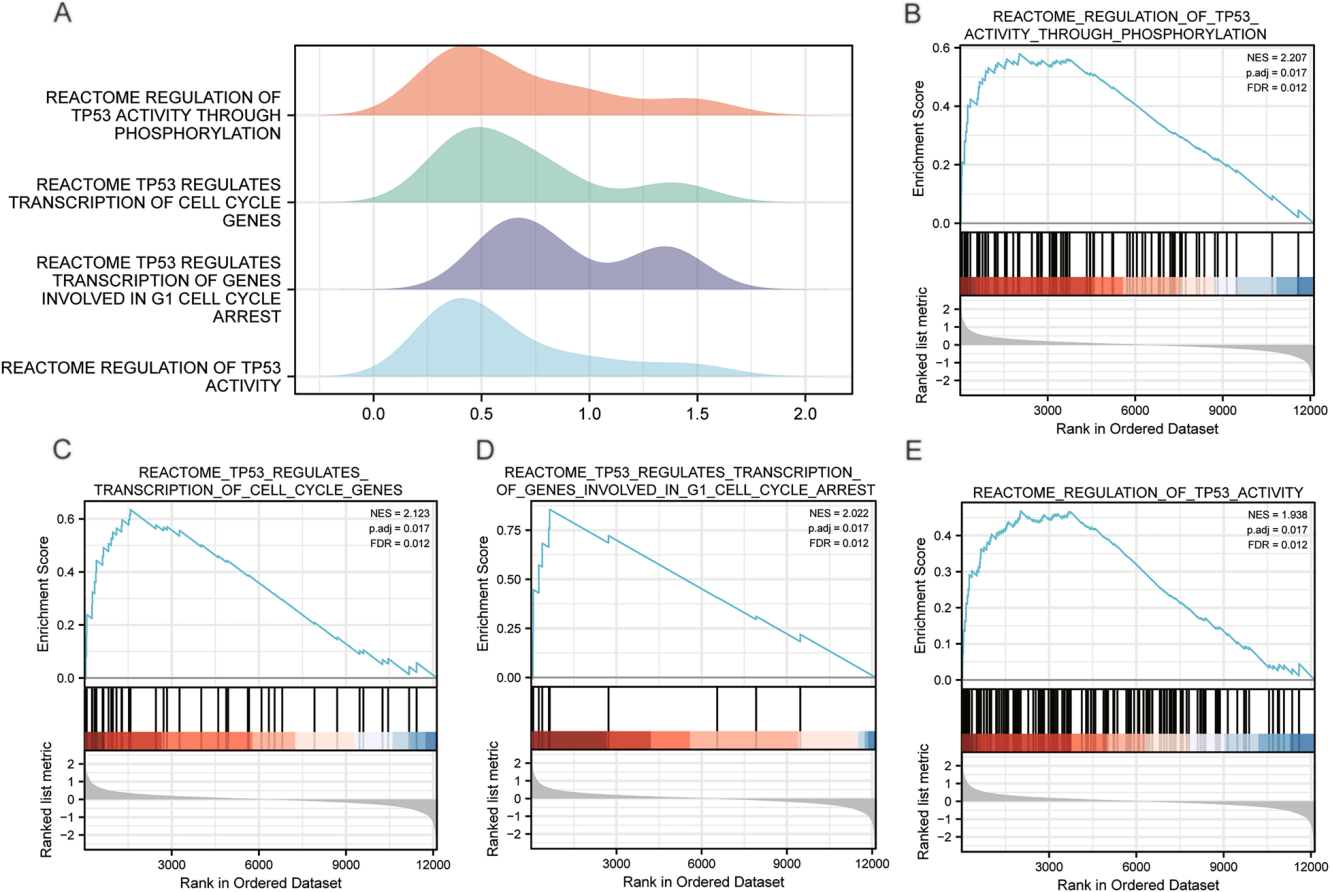
GSEA enrichment analysis of the TCGA-LIHC dataset. (**A**) The Ridge plot of GSEA analysis for Top4 biological functions in TCGA-LIHC dataset. (**B**) Visualization chart of GSEA enrichment analysis results of TCGA-LIHC genes in high and low risk groups (group: high and low) with REACTOME_ REGULATION_OF_TP53_ACTIVITY_ THROUGH_PHOSPHORYLATION, (**C**) REACTOME_TP53_REGULATES_ TRANSCRIPTION_OF_CELL_ CYCLE_GENES, (**D**) REACTOME_ TP53_REGULATES_ TRANSCRIPTION_OF_GENES_INVOLVED_IN_G1_CELL_CYCLE_ARREST, (**E**) REACTOME_ REGULATION_ OF_TP53_ ACTIVITY. GSEA: Gene Set Enrichment Analysis; NES: Normalized Enrichment Scores; FDR: false discovery rate; NES: normalized enrichment score.

The enrichment analysis revealed the association between the expression level of all genes and the biological processes, the cellular components and the molecular functions in the TCGA-LIHC dataset (Supplementary Table S3). Using *p* value < 0.05 and q value < 0.1 as screening criteria, the normalized enrichment scores (NES) top4 pathways related to TP53 were taken to plot the ridge plot (Fig. 6A). The results showed that the genes in dataset TCGA-LIHC of high and low hazard groups were significantly enriched in REACTOME_REGULATION_ OF_TP53_ACTIVITY_ THROUGH_PHOSPHORYLATION (Fig. 6B), REACTOME_TP53_REGULATES_ TRANSCRIPTION_OF_CELL_CYCLE_GENES (Fig. 6C), REACTOME_TP53_ REGULATES_TRANSCRIPTION_OF_GENES_ INVOLVED_IN_G1_CELL_ CYCLE_ARREST (Fig. 6D), REACTOME_ REGULATION_OF_TP53_ ACTIVITY (Fig. 6E).

### Construction of molecular interaction networks

**Figure 7:**
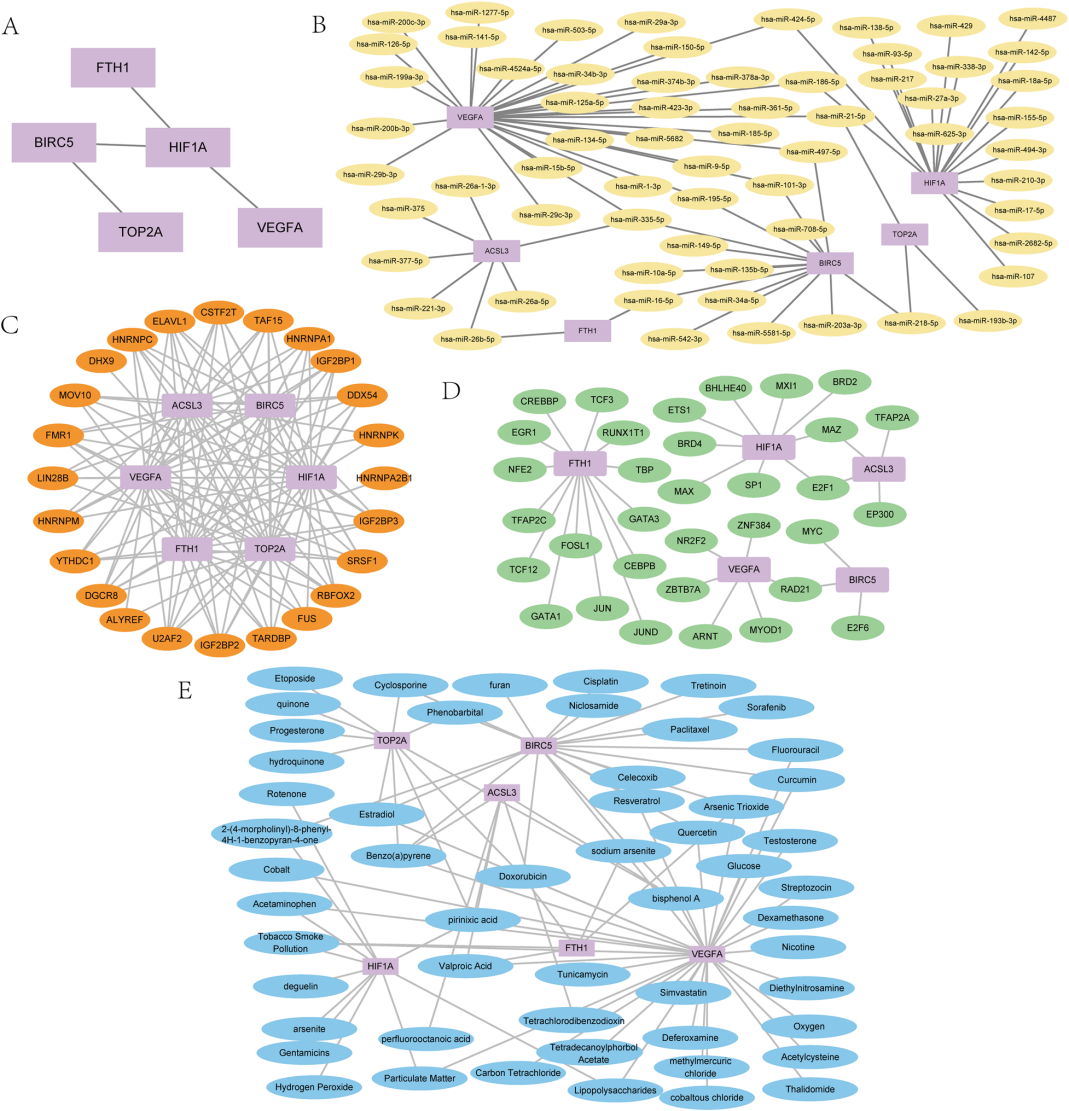
Building the molecular interaction networks (**A**) Protein–protein interaction network of differentially expressed prognosis-related ferroptosis and drug-resistant genes, (**B**) mRNA-miRNA interaction network, (**C**) mRNA-RBP interaction network, (**D**) mRNA-TF interactome network, (**E**) mRNA-drugs interaction network. PPI: Protein–protein interaction RBP: RNA binding protein; TF: Transcription factors.

PPI network (Fig. 7A) of the six gene signature showed HIF1A had the highest correlation with other genes. The highest correlation was observed between HIF1A and VEGFA with the score 0.998, followed by HIF1A and BIRC5 with the score 0.93. The correlation score between BIRC5 and TOP2A was 0.959, and the score between FTH1 and HIF1A was at 0.429 in the PPI network. However, the gene ACSL3 didn’t have any protein–protein interactions with other genes. (The specific PPI networks were shown in Supplementary Data Sheet S3).

The mRNA-miRNA interaction network was presented in Fig. 7B, and we identified a total of 75 pairs of mRNA-miRNA interactions in miRTarBase and TarBase database. Among them, the gene VEGFA had the most mRNA-miRNA interactions with a total of 31 pairs. The specific mRNA-miRNA relationships were shown in Supplementary Data Sheet S4.

The mRNA-RBP interaction network was shown in Fig. 7C. The mRNA-RBP interaction network consisted of 6 hub genes (TOP2A, BIRC5, VEGFA, HIF1A, FTH1, ACSL3), 24 RBP molecules, and a total of 120 mRNA-RBP interaction pairs. The specific mRNA-RBP interaction pairs were shown in Supplementary Data Sheet S5.

The mRNA-TF interaction network (Fig. 7D) showed the obtained five ferroptosis and drug resistance-related genes (ACSL3, BIRC5, FTH1, HIF1A, VEGFA) and 36 TFs interactions. The gene FTH1 had the most interactions with TFs of 14 pairs. The specific mRNA-TF interactions were in Supplementary Data Sheet S6.

The mRNA-drugs regulatory network (Fig. 7E) was visualized (Supplementary Data Sheet S7 showed specific mRNA-drugs relationships). A total of 54 direct and indirect drug targets with the six prognosis-related genes were obtained by CTD database, comprising a total of 87 pairs of mRNA-drug interactions. Among them, the drugs Benzo(a)pyrene, bisphenol A, Doxorubicin, and pirinixic acid had interactions with at least four ferroptosis and drug resistance-related genes.

### Validation of the differentially expressed prognosis-related genes

**Figure 8:**
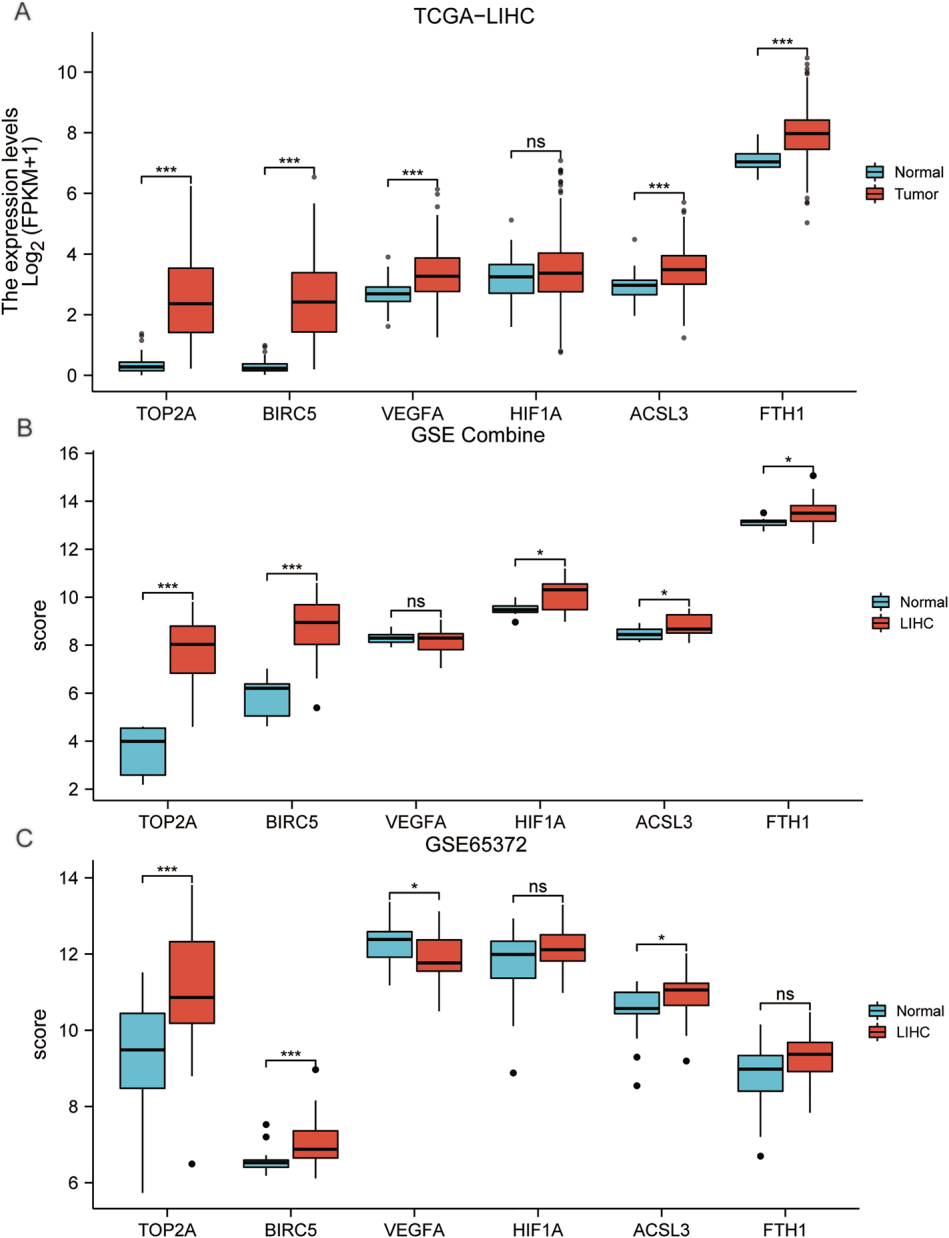
Subgroup comparison charts of differentially expressed prognosis-related ferroptosis and drug-resistant genes in different datasets. (**A**) LIHC samples (group: LIHC) and control samples (group: Normal) of TCGA-LIHC dataset, (**B**) GSE Combine dataset. (**C**) GSE65372 dataset. ns *P* ≥ 0.05, *P < 0.05, **P < 0.01, ***P < 0.001.

The group comparison chart of the gene signature showed that in the dataset TCGA-LIHC (Fig. 8A), the genes TOP2A, BIRC5, VEGFA, FTH1, ACSL3 had greater statistical significance (*P* < 0.001) and the gene HIF1A didn’t have (*P* ≥ 0.05). In the GSE Combine dataset (Fig. 8B), the genes TOP2A, BICR5 had greater statistical significance (*P* < 0.001), genes HIF1A, ACSL3, FTH1 had statistical significance (*p* < 0.05), and gene VEGFA didn’t have (*P* ≥ 0.05).In the GSE65372 dataset (Fig. 8C), genes TOP2A, BRIRC5 had greater statistical significance (*P* < 0.001), genes VEGFA, ACSL3 had statistical significance (*P* < 0.05), genes HIF1A, FTH1 didn’t have (*P* ≥ 0.05). In summary, the expression of genes TOP2A and BIRC5 in all datasets had greater statistical significance in both LIHC samples (group: LIHC) and control samples (group: Normal).

### Prognostic and clinical correlation analysis of DEGs

We visualized the risk factor groups of the LASSO regression prognostic model with a risk factor graph (Fig. S1A), which consisted of 3 parts: risk groups, survival outcome and the heatmap. The results showed that patients who died were mainly concentrated in the high-hazard group and the expression level of the hub genes was higher in the high-hazard group than the low one. The ROC for the gene signature (TOP2A, BIRC5, VEGFA, HIF1A, FTH1, ACSL3) of different clinical subgroups from the dataset TCGA-LIHC was displayed in Fig. S1B–F (The ROC diagnostic accuracy of the gene signature was shown in supplementary materials).

**Figure 9:**
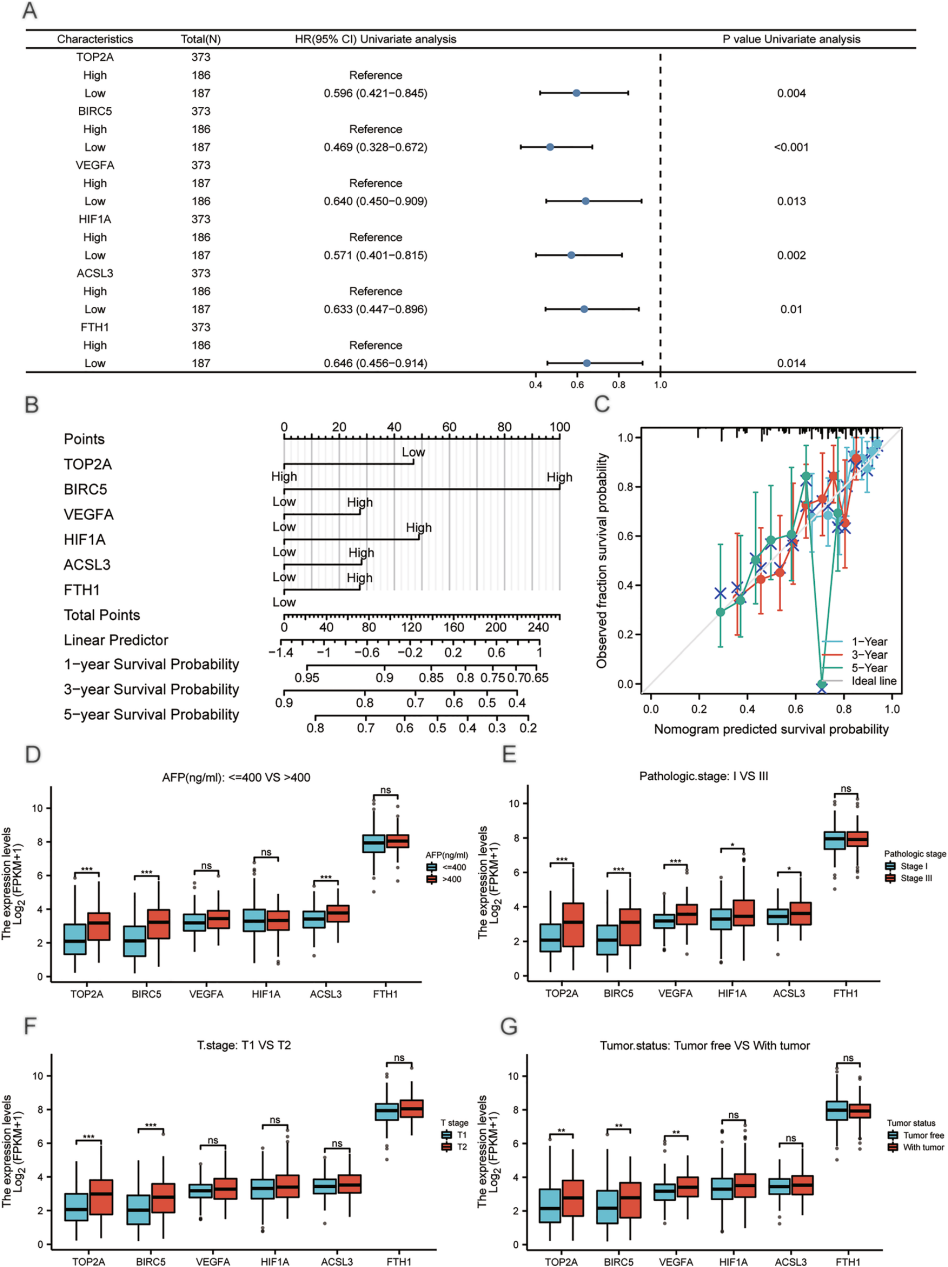
Forest plot, Alignment diagram, Calibration analysis and group comparison plots of clinical correlation. (**A**) The Forest plot of univariate cox regression analysis of differentially expressed prognosis-related ferroptosis and drug-resistant genes (**B**) Alignment diagram (**C**) 1, 3, and 5-years calibration curves. (**D**) Group comparison plots of differentially expressed prognosis-related ferroptosis and drug-resistant genes in clinical groups of AFP (ng/ml): <  = 400 and > 400, (**E**) pathologic stage: Stage I and Stage III, (**F**) T stage: T1 and T2, (**G**) tumor status: Tumor free and With tumor. ns *P* ≥ 0.05, *P < 0.05, **P < 0.01, ***P < 0.001.

Univariate and multivariate cox regression analysis in the TCGA-LIHC dataset showed the expression of gene signature was significantly associated with clinical prognosis (Supplementary Table S4). We collated the results of univariate cox regression and presented them by a forest plot (Fig. 9A), and we validated the LASSO regression model and multivariate cox regression results by nomogram analysis (Fig. 9B). Our proportional hazards model had high diagnostic accuracy for the 1, 3, and 5-years survival of HCC patients in the TCGA-LIHC dataset. The BIRC5 expression contributed more to the model than other variables. In addition, the calibration curves (Fig. 9C) showed that the proportional hazards model fitted best at predicting 1- and 3-years survival based on the gene expression level.

The group comparison chart of different clinical variables showed that in the clinical group AFP (ng/ml): < = 400 and > 400 (Fig. 9D), the genes TOP2A, BIRC5, ACSL3 had greater statistical significance, and the genes VEGFA, HIF1A, FTH1 didn’t have statistical significance. In the clinical group pathologic stage: Stage I and Stage III (Fig. 9E), the genes TOP2A, BIRC5, VEGFA had greater statistical significance, the genes HIF1A and ACSL3 had statistical significance, and the gene FTH1 didn’t have statistical significance. In the clinical group T stage: T1 and T2 (Fig. 9F), the genes TOP2A, BIRC5 had greater statistical significance and the genes VEGFA, HIF1A, FTH1, ACSL3 didn’t have statistical significance. In the clinical group Tumor status: Tumor free and with tumor (Fig. 9G), the genes TOP2A, BIRC5, VEGFA had highly statistical significance, while the genes HIF1A, ACSL3, FTH1 didn’t have statistical significance.

**Figure 10:**
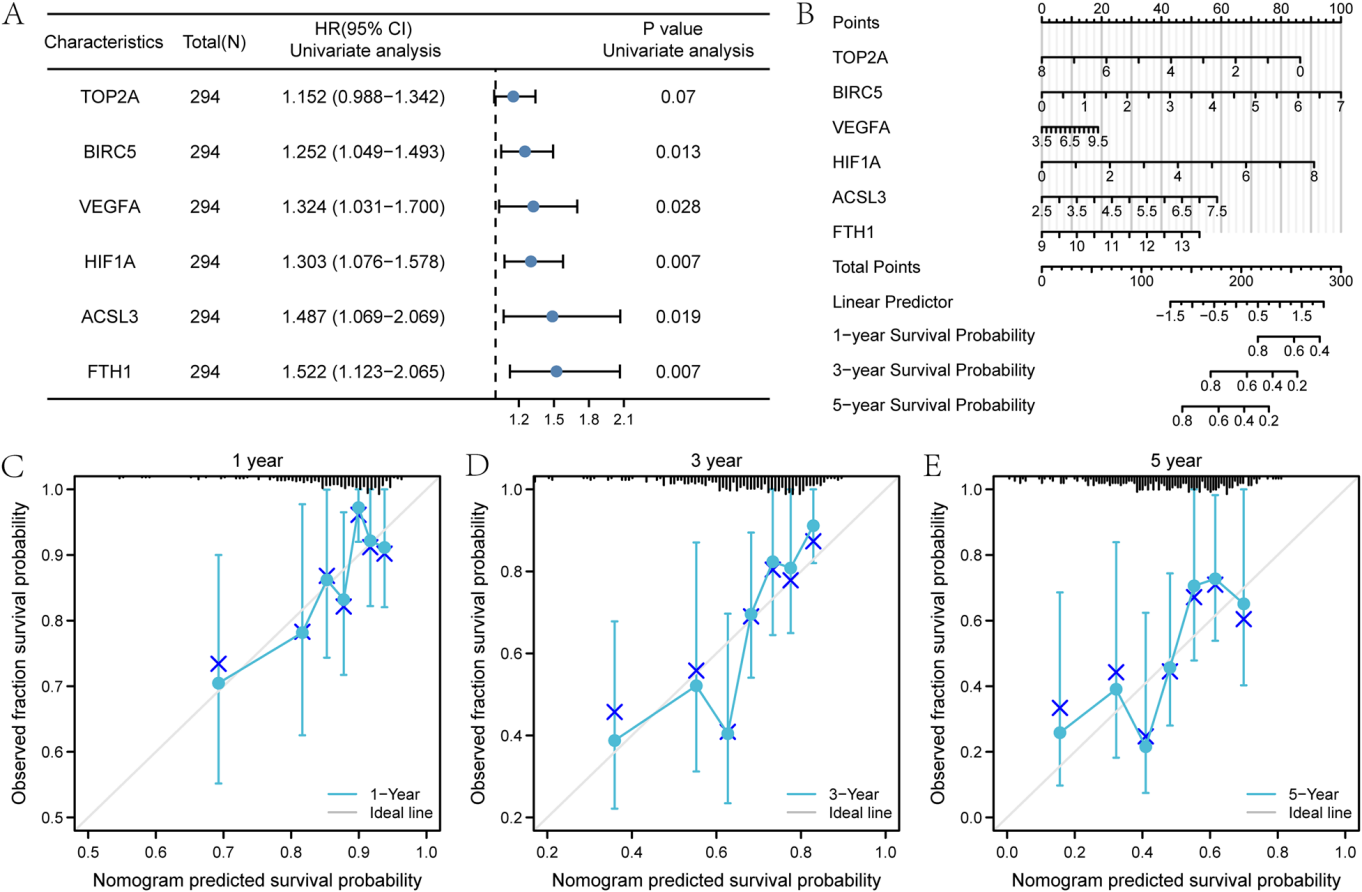
Forest plot, Alignment diagram, and calibration analysis of the LIHC-US verification dataset. **(A)** Forest plot of univariate cox regression analysis of differentially expressed prognosis-related ferroptosis and drug-resistant genes in the LIHC-US dataset. **(B)** Alignment diagram of multivariate cox regression analysis of differentially expressed prognosis-related ferroptosis and drug-resistant genes in the LIHC-US dataset. Calibration curves for 1-year **(C)**, 3-years **(D)**, and 5-years **(E)** of multivariate cox regression analysis of prognosis-related ferroptosis and drug-resistant genes in the LIHC-US dataset. ns *P* ≥ 0.05, *P < 0.05, **P < 0.01, ***P < 0.001.

Univariate and multivariate cox regression analysis in the LIHC-US dataset showed that the expression level of the six genes significantly correlates with clinical outcomes. (Supplementary Table S5). Univariate cox regression analysis was presented in a forest plot (Fig. 10A). The nomogram analysis (Fig. 10B) showed that the proportional hazards model had high accuracy for the diagnosis of 1-year, 3-years, and 5-years survival of HCC patients in LIHC-US dataset. The BIRC5, HIF1A, and TOP2A expression significantly contributed to the model, and BIRC5 was the most among all variables. The proportional hazards model and calibration analysis (Fig. 10C–E) showed that the model had the best prediction for 1-year survival time.

The proportional hazards model in the LIHC-US dataset had high similarity to the risk model in the TCGA-LIHC dataset, which could effectively validate the accuracy of our identified six genes for clinical diagnosis in HCC.

### Immune checkpoint and drug sensitivity analysis of differentially expressed prognosis-related genes

**Figure 11:**
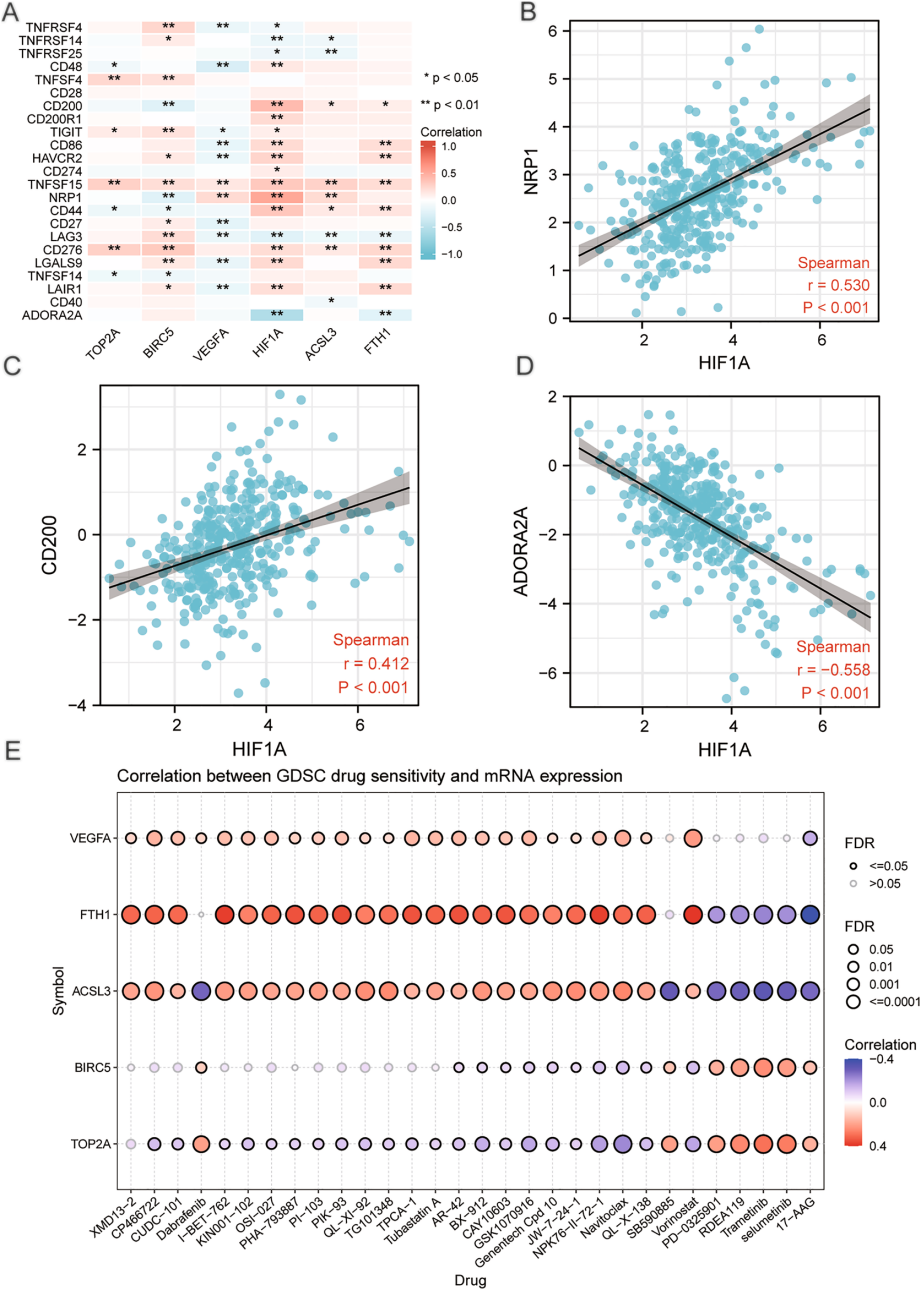
Heatmap, scatter plot and drug-sensitivity bubble plot of correlation analysis for the prognosis-related genes and immune checkpoint. (**A**)Heatmap of correlation analysis for the gene signature and immune checkpoint. (**B**) Scatter plot of correlation between gene HIF1A and immune checkpoint NRP1, (**C**) HIF1A and immune checkpoint CD200, (**D**) HIF1A and immune checkpoint ADORA2A. (**E**) Drug-sensitivity bubble plot for the correlation between gene VEGFA, FTH1, ACSL3, BIRC5, TOP2A and drug IC50. FDR: false discovery rate. ns *P* ≥ 0.05, *P < 0.05, **P < 0.01, ***P < 0.001.

We extracted the expression profile data from the TCGA-LIHC dataset for 44 immune checkpoints obtained through the relevant literature, and 23 immune checkpoints (TNFRSF4, TNFRSF14, TNFRSF25, CD48, TNFSF4, CD28, CD200, CD200R1, TIGIT, CD86. HAVCR2, CD274, TNFSF15, NRP1, CD44, CD27, LAG3, CD276, LGALS9, TNFSF14, LAIR1, CD40, ADORA2A) were matched in the TCGA-LIHC dataset. We calculated the correlation matrices of these 23 immune checkpoints and the genes (TOP2A, BIRC5, VEGFA, HIF1A, FTH1, ACSL3) , and correlation heatmaps were plotted to visualize the results (Fig. 11A). The correlation scatter plots showed the six gene expression was significantly correlated with the immune checkpoints (*P* < 0.05). Among them, the gene HIF1A had moderate positive correlation with the immune checkpoint NRP1 (r = 0.530, *P* < 0.001, Fig. 11B) and CD200 (r = 0.412, *P* < 0.001, Fig. 11C). The gene HIF1A had moderate negative correlation with the immune checkpoint ADORA2A (r = − 0.588, *P* < 0.001, Fig. 11D).

Correlations between the gene signature and the drug IC50 were visualized by the bubble plot (Fig. 11E). The results showed that gene VEGFA, FTH1 and ACSL3 had positive correlation with most of the drug IC50, and gene TOP2A had negative correlation with most of the drug IC50.

### The expression level of six prognosis-related ferroptosis and drug-resistant genes

**Figure 12:**
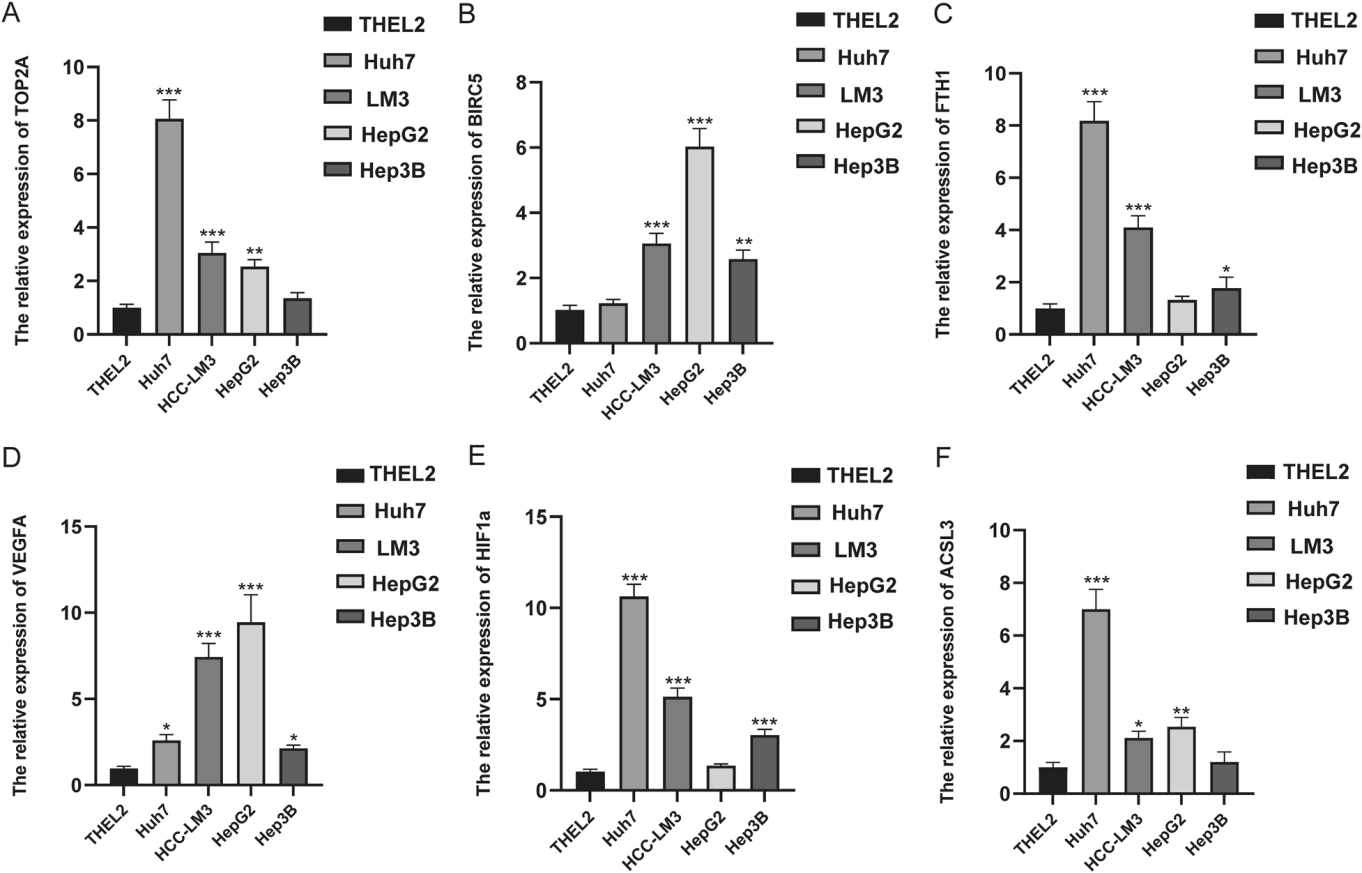
The expression level of six prognosis-related ferroptosis and drug-resistant genes in THEL2, Huh7, HCC-LM3, HepG2, and Hep3B cell lines. Expression of TOP2A **(A)**, BIRC5 **(B)**, FTH1 **(C)**, VEGFA **(D)**, HIF1A **(E)** and ACSL3 **(F)** in normal hepatocyte (THEL2) and hepatocellular carcinoma cells (Huh7, HCC-LM3, HepG2, Hep3B) detected by RT-qPCR. *p < 0.05, **p < 0.01, ***p < 0.001.

To further verify the expression of these screened hub genes in HCC, we detected the expression of our signature in HCC cell lines on mRNA level in contrast to the immortalized human hepatocyte THEL2. Total RNAs of Hep3B, HepG2, Huh7, HCC-LM3 and THEL2 were extracted and real-time quantitative PCR was conducted. We found that the overall expression of the six hub genes was higher in HCC cells compared with that in normal hepatocyte THEL2 (Fig. 12A–F), which was basically consistent with our database analysis results.

Other results can be found in the supplementary materials.

## Discussion

While the range of approaches to HCC treatment is broadening, there are novel avenues available for treating HCC that differ from traditional treatments. The advent of tumor immunotherapy and molecular-targeted therapies provide new opportunities for HCC treatment. Nonetheless, these therapies remain limited for advanced HCC due to the high incidence of relapse and metastasis. Hence, it is necessary to find new molecular targets for early diagnosis and treatment of HCC to improve the prognosis of patients. Ferroptosis is an iron-dependent form of regulatory cell death caused by excessive lipid peroxidation, which is associated with the occurrence and therapeutic response of various types of tumors. Increasing evidence has suggested that activating ferroptosis may effectively inhibit tumor growth, whereas ferroptosis inducers may overcome the disadvantages of traditional chemotherapeutic agents^31^.However, there was no comprehensive and independent analysis of the gene prognostic model related to ferroptosis and drug resistance in HCC. Our current study constructed a new gene signature related to ferroptosis and drug resistance to predict the clinical prognosis. In addition, the study explored possible molecular mechanisms to provide new ideas for clinical practice in HCC.

As the mechanism of ferroptosis has been investigated, ferroptosis-related genes (FRGs) have been established and applied to predict the prognosis of HCC patients though they played different roles in function^20,22,32^. Nonetheless, our gene signature provides a more solid clinical reference in HCC by combining ferroptosis and drug- resistant phenotypes in a comprehensively prognosis-related analysis, which had a better predictive ability. Regarding the studies of drug resistance-related gene signature and pathways in HCC patients, Jiang, et al. identified seven promising therapeutic agents and 13 hub genes related to sorafenib resistance in HCC by integrated transcriptomic analysis^33^. In addition, a great many miRNAs were directly involved in drug resistance in cancer cells^34^. So finding and identifying drug resistance-related genes and exploring their mechanisms in formation will solve the thorny problems in advanced HCC treatment. Unlike previous studies, we comprehensively analyzed the hub genes related to ferroptosis and drug resistance and built a prognostic model more effective to predict the prognosis in HCC patients. Moreover, we have identified their common enriched pathways and functions, related molecule interactions, and clinical variables in liver cancer. It is the first initiative in this field, the findings will provide a deeper understanding of the molecular studies and underlying mechanisms involved in ferroptosis and chemotherapy resistance.

More evidence has supported the important role of our gene signature in drug resistance to hepatocellular carcinoma. For example, TOP2A is a cellular topoisomerase that determines the response of tumor cells to chemotherapy, and the chemosensitivity of tumor cells can be determined by intracellular topoisomerase level^35^. In recent years, it has been found that both TOP2A mRNA and protein were significantly expressed in HCC patients^36^. Meanwhile, Wang, et al. found that when the cells were exposed to Etoposide (a TOP2A toxin), miR-23a inhibited topoisomerase TOP1 expression, thereby enhancing drug sensitivity in HCC cells^37^. This suggests TOP2A is a potential target for regulating drug sensitivity to chemotherapeutics in HCC. In addition, BIRC5 encodes a survivin protein which is considered to be the most powerful inhibitor of apoptosis^38^. High expression of BIRC5 was observed in HCC cells and tissues^39^. While in rat hepatoma cell lines, BIRC5 expression promoted resistance to cisplatin-induced apoptosis through PI3K-dependent survivin expression^40^. BIRC5-specific siRNA inhibited HCC cell growth and reversed drug resistance by inhibiting lung resistance-related protein (LRP) so that improved chemotherapy sensitivity in vitro and in vivo^41^.VEGFA is the most powerful Vascular endothelial growth factor (VEGF) subtype and the mechanisms involved in VEGF regulating in HCC included the HIF1-α pathway, HBx protein activation, tumor suppressor gene loss or inactivation, and multiple signal transduction pathways^42^. Studies have shown that VEGF inhibition can overcome resistance to immune checkpoint inhibition in advanced HCC patients and be more effective against tumor activity^43^. Also, patients with VEGF amplification were more sensitive to sorafenib in HCC^44^.

The rapid proliferation of HCC cells and the abnormal structure and function of tumor vascular lead to a hypoxic environment, which upregulates the hypoxia-inducible factors (HIFs) to respond. As a subunit of the hypoxia-inducible factor-1 (HIF1), HIF1A was found highly expressed in HCC tissue, suggesting that HCC patients may have a late stage and poor prognosis^45^. Moreover, Wei, et al. found that hypoxia promoted the recruitment of HIF-1α to VEGF promoter, and activated VEGF transcription^46^. Consistently, our PPI network results showed that the correlation between HIF1A and VEGFA was strong, with a score of 0.998. Bai, et al. investigated that in HeLa cells of cervical cancer, hypoxia upregulated the expression of both HIF-1α and BIRC5, and BIRC5 transcription was activated and correlated with overexpression of HIF-1α^47^. Similarly, our PPI network showed a high correlation score of HIF1A and BIRC5 (0.93). As the HIF-1α involved in various hypoxia-related diseases, Jin, et al. demonstrated FTH1 suppressed the transcriptional activity of HIF-1α in HCT116 cells of human colorectal cancer, and downregulated the expression of HIF-1 target genes including VEGF, CA9, and GLUT1^48^. Ferritin (FT) is a protein complex that consists of multiple repeating subunits of the FT heavy chain (FTH1) and the FT light chain (FTL), both of which are mainly involved in the uptake and release of iron^49^. Compared with normal liver tissue, FTH1 is highly expressed in HCC ^50^. As a part of the resistant gene markers, FTH1 expression was negatively correlated with chemotherapy resistance to SN38 (the active metabolite of the topoisomerase inhibitor irinotecan) and could predict the survival of patients with colorectal cancer^51^. Although reduced FTH1 expression could lead to iron overload and oxidative damage in hepatocytes^52^, there were no studies on the specific mechanism of FTH1 in regulation of HCC and its impact on anti-tumor drug chemotherapy resistance.

ACSL3 is a major regulator of ferroptosis by activating exogenous monounsaturated fatty acids in place of polyunsaturated fatty acids and blocking lipid ROS accumulation^53^. High expression level of ACSL3 increased the sensitivity of NSCLC lung cancer cells to simvastatin treatment^54^. Though studies have shown that ACSL3 expression was increased in HCC and similar to that in liver metastases, which can be used to distinguish different types of liver tumors^55^, the role of ACSL3 in liver malignant tumors is less studied and remains unclear. As a major regulator of ferroptosis and an important component mediating fatty acid metabolism, ACSL3 remains unknown for HCC treatment. We infer that ACSL3 may become a potential therapeutic target.

Our results indicated that the gene signature was significantly associated with overall survival in HCC patients, and their high expression was related to poor prognosis. The expression of TOP2A and BIRC5 had high accuracy for HCC diagnosis and was highly significant with clinical relevance. The genes VEGFA, FTH1, and ACSL3 had accuracy with the best diagnosis at 1-year, which indicates our model should be an effective tool for predicting the prognosis of HCC patients. In both the testing set (TCGA-LIHC dataset) and the validation set (LIHC-US dataset), the proportion regression risk model of the gene signature had high accuracy in diagnosing the 1-year, 3-year, and 5-year survival of HCC patients with the best fitting effect at 1-year and 3-year survival. Functional enrichment analysis revealed that ferroptosis, platinum drug resistance, HIF-1 and TP53 were the main signaling pathways. Furthermore, several previous studies have demonstrated our findings. For example, Liu et al.^56^ found that MDM4 and TOP2A bound to each other and upregulated TOP2A protein at the translation level, inhibiting p53 and increasing tumor cell proliferation. Furthermore, in the respect of tumor chemotherapy resistance, progression-free survival (PFS) was significantly correlated with the TOP2A gene increases in platinum-resistant/refractory epithelial ovarian cancer (EOC)^57^. In addition, the status of the gene BIRC5 affected prognosis and chemotherapy sensitivity in epithelial ovarian cancer^58^, and may be related to platinum resistance. In the hypoxia and drug resistance of tumor treatment, Gong et al.^59^ found the knockdown of kruppel-like factor 5, which promoted hypoxia-induced cell apoptosis by directly regulating HIF-1α expression in hypoxia-mediated cisplatin-resistant non-small cell lung cancer (NSCLC) cells. In our molecular interaction networks, Xu and Ghosh et al.^60,61^ demonstrated the interactions between the miRNAs and target genes, which were consistent with our predicted findings. All the above results were consistent with our functional enrichment and molecular interaction networks results, indicating that our prognostic model is highly effective.

As immunotherapy has become the new standard for the treatment of advanced liver cancer worldwide^62^. However, only a small percentage of HCC patients have a good response to immunotherapy. Thus, choosing the appropriate individualized targeted therapy remains a challenge for HCC patients. Interestingly, the results provided a new perspective. In our model, the genes VEGFA, FTH1 and ACSL3 had positive correlations with the IC50 of most drugs, while the gene TOP2A had a negative correlation with the IC50 of most drugs. The gene HIF1A had a moderate positive correlation with the immune checkpoint NRP1and CD200, but the gene HIF1A had a moderate negative correlation with the immune checkpoint ADORA2A. It is suggested that immune checkpoint inhibitors are more effective in HCC patients with HIF1A as a risk feature. Our findings suggest that treatment of targeting the hub genes may open a new chapter in cancer immunotherapy.

Based on the gene signature identified in this study, we planned to summarize the clinical relevance and prognostic value of the signature through a large retrospective analysis. Furthermore, we aimed to investigate its regulatory modes and molecular mechanisms in the pathogenesis of HCC in cellular and animal models, to analyze their downstream pathways and targets, and thus to explore their clinical value. The prognostic model we established will be evaluated for its correlation with clinical outcomes using immunohistochemistry and RNA sequencing analysis, and the gene expression changes will be assessed with survival improvement trends of patients who received chemotherapy. Prospective clinical trials will be conducted to provide the highest level of evidence for the efficacy of our clinical biomarkers.

Our results provide a new visual for effective HCC clinical diagnosis and therapeutic strategies, as well as a theoretical basis for future study. There are still some limitations in this study. First, we used data from public databases to construct and validate the prognostic model, so some deviations were inevitable. Second, the clinical significance of our model needs to be further evaluated by real prospective data. Moreover, in vivo and in vitro experiments are needed to reveal their molecular mechanisms in HCC. Third, the relationship between other modes of cell death and drug resistance in HCC remains poorly understood. Thus, further research is needed to explore the relationship. Finally, in HCC, the potential mechanisms between these hub genes of ferroptosis and tumor multidrug resistance are unclear and deserve further exploration.

## Conclusion

In conclusion, we found and verified a new gene signature related to ferroptosis and drug resistance with a high capacity to predict the clinical prognosis of HCC. Furthermore, we identified several innovative biomarkers and molecular mechanisms for diagnostic and therapeutic targets of HCC by comprehensively analyzing the biological function and pathways, molecular interaction networks, immune checkpoints, and drug sensitivity. These findings provide a novel perspective for the diagnosis and treatment of HCC.

## Data availability 

All datasets used in this work are publicly available. The TCGA-LIHC dataset is available at the weblink (https://portal.gdc.cancer.gov/projects/TCGA-LIHC) and data acquired at the weblink (https://portal.gdc.cancer.gov/repository?facetTab=files&filters=%7B%22content%22%3A%5B%7B%22content%22%3A%7B%22field%22%3A%22cases.project.project_id%22%2C%22value%22%3A%5B%22TCGA-LIHC%22%5D%7D%2C%22op%22%3A%22in%22%7D%2C%7B%22content%22%3A%7B%22field%22%3A%22files.data_category%22%2C%22value%22%3A%5B%22Transcriptome%20Profiling%22%5D%7D%2C%22op%22%3A%22in%22%7D%5D%2C%22op%22%3A%22and%22%7D&searchTableTab=files).Microarray datasets are available through NCBI’s Gene Expression Omnibus (GEO, http://www.ncbi.nlm.nih.gov/geo/) database, which were GSE6222, GSE46408 and GSE65372 datasets. Dataset GSE6222 is available at the weblink https://www.ncbi.nlm.nih.gov/geo/query/acc.cgi?acc=GSE6222 and was generated in Liao et al. Dataset GSE46408 is available at the weblink https://www.ncbi.nlm.nih.gov/geo/query/acc.cgi?acc=GSE46408 and was generated in Chen et al. Dataset GSE65372 is available at the weblink https://www.ncbi.nlm.nih.gov/geo/query/acc.cgi?acc=GSE65372 and was generated in Sia et al. The LIHC-US dataset is available at the weblink (https://dcc.icgc.org/) and data acquired at the weblink (https://dcc.icgc.org/search?filters=%7B%22donor%22:%7B%22primarySite%22:%7B%22is%22:%5B%22Liver%22%5D%7D,%22analysisTypes%22:%7B%22is%22:%5B%22RNA-Seq%22%5D%7D,%22projectId%22:%7B%22is%22:%5B%22LIHC-US%22%5D%7D%7D%7D&donors=%7B%22from%22:1%7D).

## Supplementary Information

Supplementary Information.
