# Supplementary material for: Identification of ferroptosis and drug resistance related hub genes to predict the prognosis in Hepatocellular Carcinoma
Source: Sci Rep. 2023 May 29;13:8681. doi: 10.1038/s41598-023-35796-z (PMC10227089; doi:10.1038/s41598-023-35796-z)
Supplement: Supplementary file 1 — Supplementary Information. [file 41598_2023_35796_MOESM1_ESM.zip › supplementary information/supplementary materials..docx]

Identification of ferroptosis and drug resistance related hub genes to predict the prognosis in Hepatocellular Carcinoma

Chengjun Li^1^, Xiaomeng Cui^1^, Yarui Li^1^, Dan Guo^1^, Shuixiang He^1*^

^1^Department of Gastroenterology, the First Affiliated Hospital of Xi’an Jiaotong University, Xi’an, Shaanxi, China

*** Correspondence:**Shuixiang He
dyyyjxk@mail.xjtu.edu.cn.

**Supplementary Section**

**Prognostic analysis of DEGs and clinical correlation analysis**

The ROC for the gene signature (TOP2A, BIRC5, VEGFA, HIF1A, FTH1, ACSL3) in different clinical information subgroups from the dataset TCGA-LIHC displayed that the genes TOP2A (AUC=0.973) and BIRC5 (AUC=0.981) had higher accuracy in the diagnosis of HCC patients and normal patients (FigureS1.B), the genes VEGFA (AUC=0.731), ACSL3 (AUC=0.753), FTH1 (AUC=0.862) had some accuracy and the gene HIF1A (AUC=0.563) had lower accuracy. In the diagnosis of HCC patients N. stage: N0 and N1 (FigureS1.C), the genes VEGFA (AUC=0.769), ACSL3 (AUC=0.713) had some accuracy, the genes TOP2A (AUC=0.608), HIF1A (AUC=0.629), FTH1 (AUC=0.506) had lower accuracy, and the gene BIRC5 (AUC=0.495) had no accuracy.

In the diagnosis of HCC patients pathologic stage: Stage III and Stage IV (FigureS1.D), the genes TOP2A (AUC=0.772), BIRC5 (AUC=0.725) had some accuracy, the genes VEGFA (AUC=0.631), FTH1 (AUC= 0.652) had lower accuracy and the genes HIF1A (AUC=0.471), ACSL3 (AUC=0.494) didn’t have accuracy. In the diagnosis of HCC patients M.stage: M0 and M1 (FigureS1.E), the gene FTH1 (AUC= 0.739) had some accuracy, the genes TOP2A (AUC= 0.674), BIRC5 (AUC= 0.548), VEGFA (AUC= 0.526), ACSL3 (AUC= 0.622) had lower accuracy and the gene HIF1A (AUC=0.470) didn’t have accuracy. In the diagnosis of HCC patients T.stage: T1 and T4 (FigureS1.F), the genes BIRC5 (AUC=0.742), ACSL3 (AUC=0.720) had some accuracy, the genes TOP2A (AUC=0.683), VEGFA (AUC=0.601), HIF1A (AUC= 0.638), FTH1 (AUC=0.697) had lower accuracy.


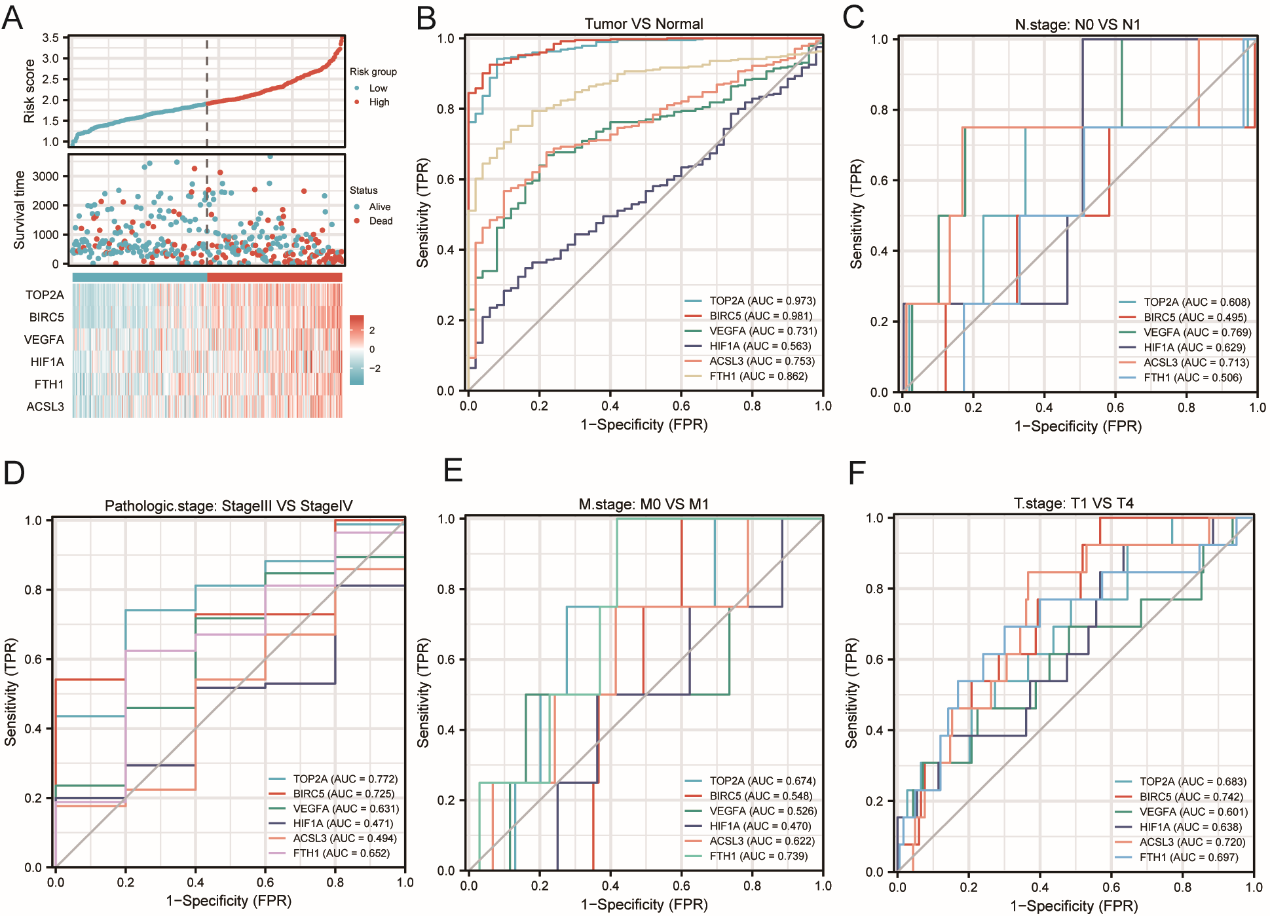


**Figure S1.** Risk factor graph and ROC curves for different clinical variables. (**A**) Risk factor graph for differentially expressed prognosis-related ferroptosis and drug resistant genes in the constructed LASSO regression prognostic model. (**B**) ROC curves for HCC (LIHC) and control (Normal) groups. (**C**) ROC curves for the N stage: N0 and N1 groups. (**D**) ROC curves for pathologic stage: Stage III and Stage IV groups. (**E**) ROC curves for M stage: M0 and M1 groups. (**F**) ROC curves for T stage: T1 and T4 groups.

**Immunohistochemistry of differentially expressed prognosis-related genes and their expression among different single-cell subsets in HCC**

We used the human protein atlas database (HPA, www.proteinatlas.org/) to search for anatomical and structural characteristics of differentially expressed prognosis-related ferroptosis and drug resistant genes in HCC tissue versus normal tissue. Then we used the HPA database to obtain the expression of differentially expressed prognosis-related ferroptosis and drug resistant genes among different single-cell subsets in HCC and visualized the results.

The HPA database showed the results of gene expression among different HCC single-cell subsets, in which the gene TOP2A, BIRC5 had higher expression in T-cells and Erythroid cells than other HCC cell subsets (FigureS2.A-B). The gene VEGFA had higher expression in Hepatocytes and Cholangiocytes than other HCC cell subsets (FigureS2.C). The gene HIF1A had higher expression in Endothelial cells, Kupffer cells and Hepatic stellate cells than other HCC cell subsets (FigureS2.D). The gene ACSL3 had higher expression in Hepatocytes, Kupffer cells and Erythroid cells than other HCC cell subsets (FigureS2.E). The gene FTH1 had higher expression in Kupffer cells and Hepatocytes than other HCC cell subsets (FigureS2.F).

In HPA database, we obtained the gene TOP2A (FigureS2.G), BIRC5 (FigureS2.H) and gene HIF1A (FigureS2.I) in HCC tissues versus normal tissues samples. The expression levels of gene TOP2A and gene BIRC5 were higher in HCC than the normal group, and the expression level of gene HIF1A was partially higher than the normal group.


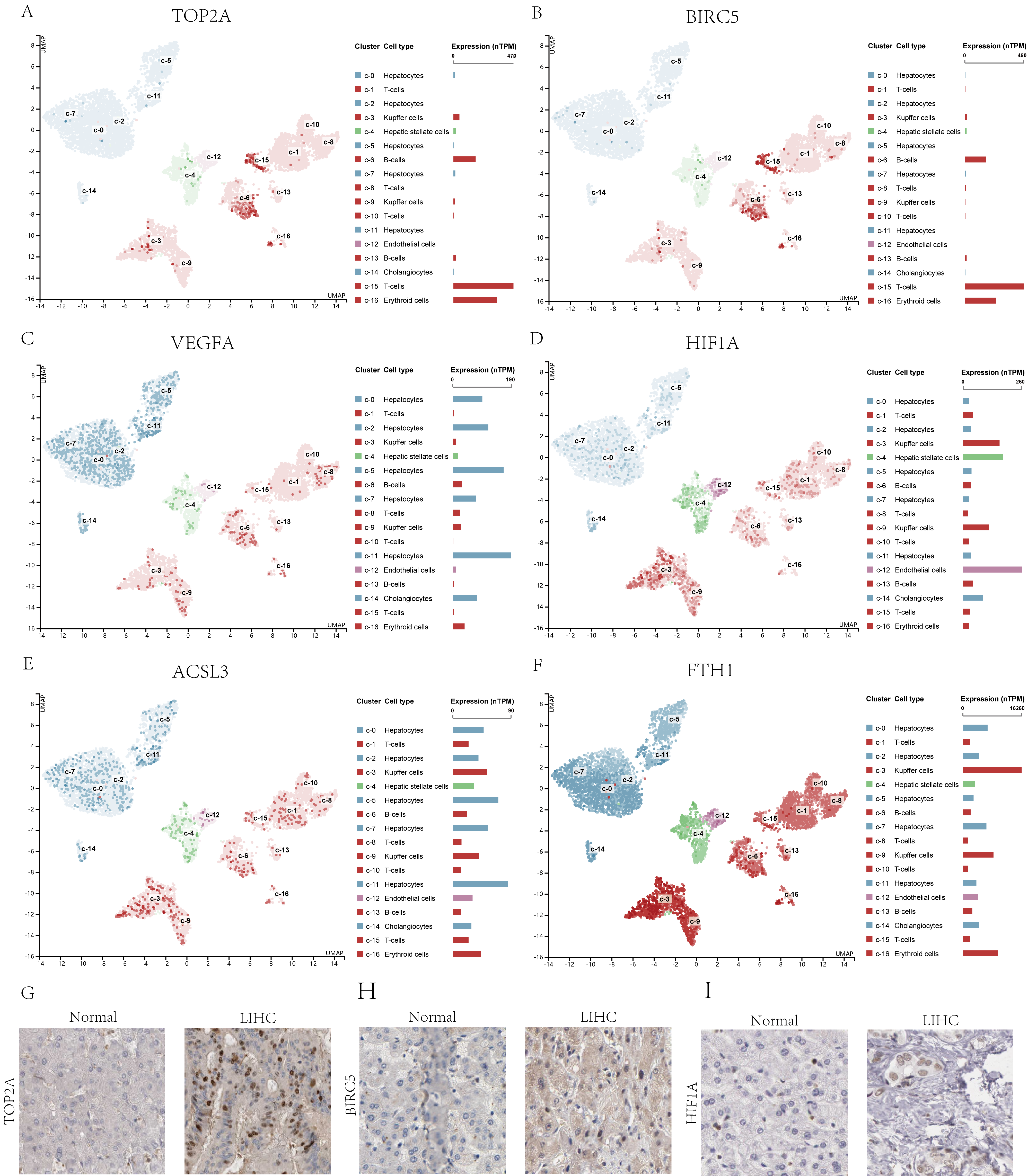


**Figure S2.** Expression of differentially expressed prognosis-related genes between different single cell subsets and HCC tissue versus normal tissue. (**A-F**) Expression of gene TOP2A, BIRC5, VEGFA, HIF1A, ACSL3, FTH1 between different single cell subsets in HCC. (**G-I**) Gene TOP2A, BIRC5, HIF1A expression in HCC tissues and normal tissues.

**Construction of the prognostic model for genes related to ferroptosis and drug resistance**

The ROC curves of 8 genes in the TCGA-LIHC dataset displayed that the genes TOP2A (AUC=0.973, FigureS3.D) and BIRC5 (AUC=0.981, FigureS3.A) had high accuracy in the diagnosis of HCC. The genes VEGFA (AUC=0.731, FigureS3.D), FTH1 (AUC=0.862, FigureS3.B), ACSL3 (AUC=0.753, FigureS3.A) and SLC7A11 (AUC=0.893. FigureS3.C) were found to be accurate for the diagnosis of HCC. The genes HIF1A (AUC=0.563, FigureS3.C) and CBS (AUC=0.595, FigureS3.B) had lower accuracy for the diagnosis of HCC.

Time-dependent ROC curves for 1 year, 3 years and 5 years (FigS3.E-L) showed that the gene BIRC5 (FigS3.F), SLC7A11 (FigS3.J), and TOP2A (FigS3.K）had higher diagnostic efficacy for HCC, with the best efficacy at 1 year. The gene ACSL3 (FigS3.E, CBS (FigS3.G), FTH1 (FigS3.H), HIF1A (FigS3.I), VEGFA (FigS3.L) had the accuracy for the diagnosis of HCC.


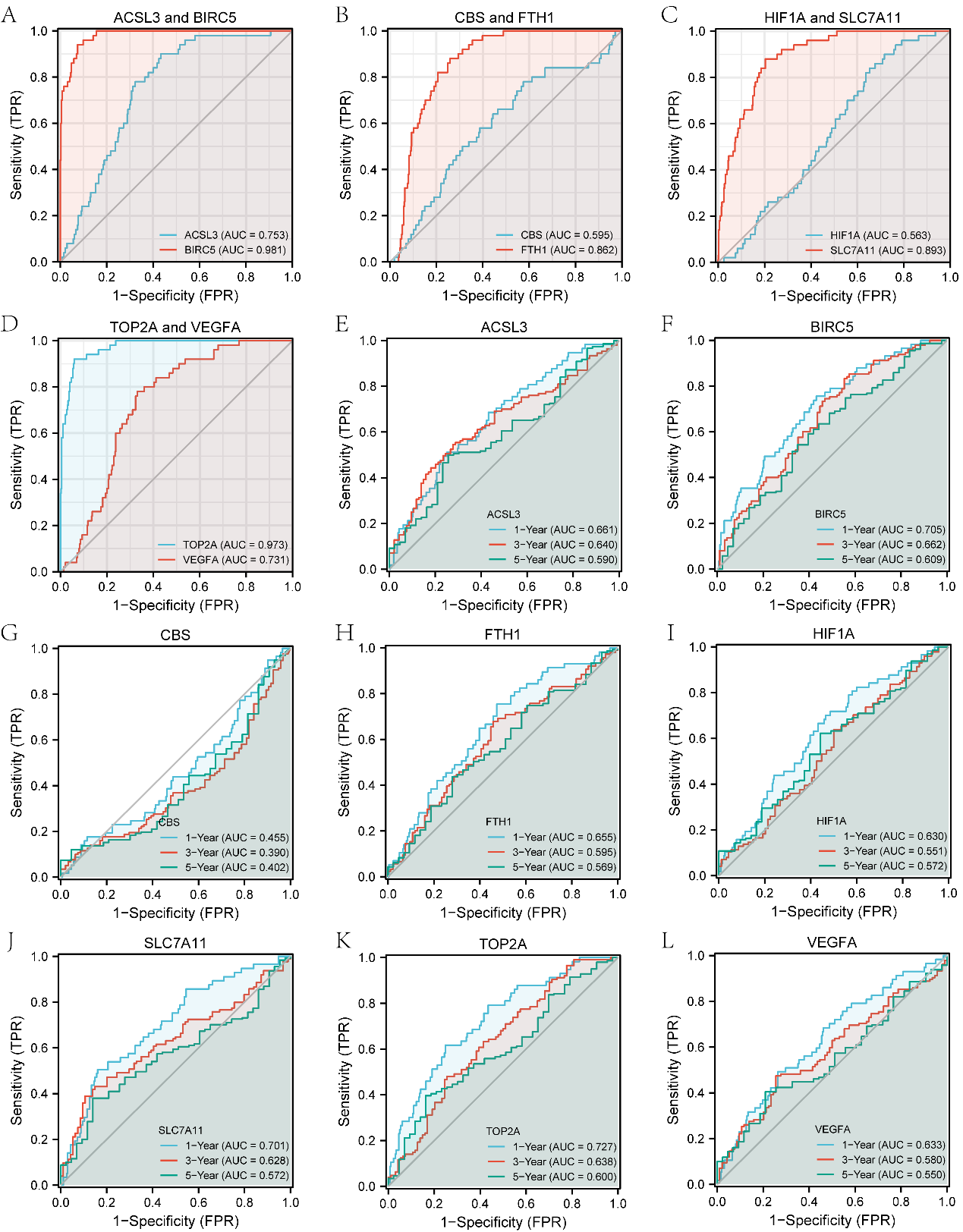


**Figure S3.** ROC curves and time-dependent ROC curves for prognosis-related ferroptosis and drug resistant genes. (**A-D**) Prognostic ROC curves of genes ACSL3, BIRC5 (A), CBS, FTH1 (B), HIF1A, SLC7A11 (C), TOP2A and VEGFA (D). (**E-L**) 1-year, 3-year, and 5-year time-dependent ROC curves of genes ACSL3 (E), BIRC5 (F), CBS (G), FTH1 (H), HIF1A (I), SLC7A11 (J), TOP2A (K), and VEGFA (L). ROC: receiver operating characteristic curve; AUC: Area Under Curve
