# Supplementary material for: Identification of ferroptosis and drug resistance related hub genes to predict the prognosis in Hepatocellular Carcinoma
Source: Sci Rep. 2023 May 29;13:8681. doi: 10.1038/s41598-023-35796-z (PMC10227089; doi:10.1038/s41598-023-35796-z)
Supplement: Supplementary file 1 — Supplementary Information. [file 41598_2023_35796_MOESM1_ESM.zip › supplementary information/Supplementary Tables/Supplementary Table S2.docx]

**Supplementary Table S2：GO and KEGG enrichment analysis results**

| ONTOLOGY | ID | Description | P value | P adjust | Q value |
| --- | --- | --- | --- | --- | --- |
| BP | GO:0060749 | mammary gland alveolus development | 8.76e-06 | 0.002 | 4.64e-04 |
| BP | GO:0061377 | mammary gland lobule development | 8.76e-06 | 0.002 | 4.64e-04 |
| BP | GO:0030949 | positive regulation of vascular endothelial growth factor receptor signaling pathway | 9.79e-06 | 0.002 | 4.64e-04 |
| BP | GO:0035162 | embryonic hemopoiesis | 1.20e-05 | 0.002 | 4.64e-04 |
| BP | GO:0002052 | positive regulation of neuroblast proliferation | 1.32e-05 | 0.002 | 4.64e-04 |
| CC | GO:0000793 | condensed chromosome | 0.001 | 0.056 | 0.017 |
| CC | GO:1904115 | axon cytoplasm | 0.014 | 0.068 | 0.021 |
| CC | GO:0005876 | spindle microtubule | 0.015 | 0.068 | 0.021 |
| CC | GO:0005778 | peroxisomal membrane | 0.015 | 0.068 | 0.021 |
| CC | GO:0031903 | microbody membrane | 0.015 | 0.068 | 0.021 |
| MF | GO:0042826 | histone deacetylase binding | 3.85e-04 | 0.022 | 0.009 |
| MF | GO:0004467 | long-chain fatty acid-CoA ligase activity | 0.004 | 0.039 | 0.016 |
| MF | GO:0005172 | vascular endothelial growth factor receptor binding | 0.004 | 0.039 | 0.016 |
| MF | GO:0005161 | platelet-derived growth factor receptor binding | 0.004 | 0.039 | 0.016 |
| MF | GO:0003996 | acyl-CoA ligase activity | 0.005 | 0.039 | 0.016 |
| KEGG | hsa05211 | Renal cell carcinoma | 7.08e-04 | 0.016 | 0.010 |
| KEGG | hsa01524 | Platinum drug resistance | 7.92e-04 | 0.016 | 0.010 |
| KEGG | hsa04066 | HIF-1 signaling pathway | 0.002 | 0.024 | 0.014 |
| KEGG | hsa05167 | Kaposi sarcoma-associated herpesvirus infection | 0.005 | 0.050 | 0.030 |
| KEGG | hsa05205 | Proteoglycans in cancer | 0.006 | 0.050 | 0.030 |

GO：Gene Ontology。BP：biological process。MF：molecular function。CC：cellular component。KEGG：Kyoto Encyclopedia of Genes and Genomes。
