# Supplementary material for: Identification of ferroptosis and drug resistance related hub genes to predict the prognosis in Hepatocellular Carcinoma
Source: Sci Rep. 2023 May 29;13:8681. doi: 10.1038/s41598-023-35796-z (PMC10227089; doi:10.1038/s41598-023-35796-z)
Supplement: Supplementary file 1 — Supplementary Information. [file 41598_2023_35796_MOESM1_ESM.zip › supplementary information/Supplementary Tables/Supplementary Table S3.docx]

**Supplementary Table S3：GSEA enrichment analysis results Top 10**

| ID | Enrichment Score | NES | p value | P adjust | Q values |
| --- | --- | --- | --- | --- | --- |
| REACTOME_TRANSPORT_OF_SMALL_MOLECULES | -0.334357859 | -1.513562241 | 0.001602564 | 0.016641284 | 0.011923011 |
| NABA_MATRISOME | -0.340271372 | -1.529893175 | 0.001610306 | 0.016641284 | 0.011923011 |
| REACTOME_METABOLISM_OF_AMINO_ACIDS_AND_DERIVATIVES | -0.481306891 | -2.106446533 | 0.001650165 | 0.016641284 | 0.011923011 |
| REACTOME_DISEASES_OF_METABOLISM | -0.450912981 | -1.860498422 | 0.001715266 | 0.016641284 | 0.011923011 |
| WP_NUCLEAR_RECEPTORS_METAPATHWAY | -0.467469606 | -1.968777586 | 0.001715266 | 0.016641284 | 0.011923011 |
| REACTOME_METABOLISM_OF_VITAMINS_AND_COFACTORS | -0.47869631 | -1.951212434 | 0.00172117 | 0.016641284 | 0.011923011 |
| REACTOME_PROTEIN_LOCALIZATION | -0.490281022 | -1.988525475 | 0.001745201 | 0.016641284 | 0.011923011 |
| REACTOME_THE_CITRIC_ACID_TCA_CYCLE_AND_RESPIRATORY_ELECTRON_TRANSPORT | -0.400542927 | -1.624557707 | 0.001745201 | 0.016641284 | 0.011923011 |
| REACTOME_BIOLOGICAL_OXIDATIONS | -0.693916266 | -2.742311529 | 0.001751313 | 0.016641284 | 0.011923011 |
| REACTOME_FATTY_ACID_METABOLISM | -0.609535534 | -2.420079258 | 0.001751313 | 0.016641284 | 0.011923011 |

GSEA：Gene Set Enrichment Analysis。LIHC：liver hepatocellular carcinoma。
