# Supplementary material for: Identification of ferroptosis and drug resistance related hub genes to predict the prognosis in Hepatocellular Carcinoma
Source: Sci Rep. 2023 May 29;13:8681. doi: 10.1038/s41598-023-35796-z (PMC10227089; doi:10.1038/s41598-023-35796-z)
Supplement: Supplementary file 1 — Supplementary Information. [file 41598_2023_35796_MOESM1_ESM.zip › supplementary information/Supplementary Tables/Supplementary Table S4.docx]

**Supplementary Table S4：Univariate/multivariate Cox regression in the TCGA-LIHC dataset**

| Characteristics | Total(N) | Univariate analysis | |  | Multivariate analysis | |
| --- | --- | --- | --- | --- | --- | --- |
|  |  | Hazard ratio (95% CI) | P value |  | Hazard ratio (95% CI) | P value |
| TOP2A | 373 |  |  |  |  |  |
| High | 186 | Reference |  |  |  |  |
| Low | 187 | 0.596 (0.421-0.845) | **0.004** |  | 1.586 (0.928-2.712) | 0.092 |
| BIRC5 | 373 |  |  |  |  |  |
| High | 186 | Reference |  |  |  |  |
| Low | 187 | 0.469 (0.328-0.672) | **<0.001** |  | 0.374 (0.226-0.619) | **<0.001** |
| VEGFA | 373 |  |  |  |  |  |
| High | 187 | Reference |  |  |  |  |
| Low | 186 | 0.640 (0.450-0.909) | **0.013** |  | 0.763 (0.523-1.114) | 0.161 |
| HIF1A | 373 |  |  |  |  |  |
| High | 186 | Reference |  |  |  |  |
| Low | 187 | 0.571 (0.401-0.815) | **0.002** |  | 0.618 (0.410-0.932) | **0.022** |
| ACSL3 | 373 |  |  |  |  |  |
| High | 186 | Reference |  |  |  |  |
| Low | 187 | 0.633 (0.447-0.896) | **0.010** |  | 0.759 (0.524-1.098) | 0.143 |
| FTH1 | 373 |  |  |  |  |  |
| High | 186 | Reference |  |  |  |  |
| Low | 187 | 0.646 (0.456-0.914) | **0.014** |  | 0.764 (0.534-1.091) | 0.138 |
