# Supplementary material for: Identification of ferroptosis and drug resistance related hub genes to predict the prognosis in Hepatocellular Carcinoma
Source: Sci Rep. 2023 May 29;13:8681. doi: 10.1038/s41598-023-35796-z (PMC10227089; doi:10.1038/s41598-023-35796-z)
Supplement: Supplementary file 1 — Supplementary Information. [file 41598_2023_35796_MOESM1_ESM.zip › supplementary information/Supplementary Tables/Supplementary Table S5.docx]

**Supplementary Table S5：Univariate/multivariate Cox regression in the LIHC-US dataset**

|  | Total(N) | Univariate analysis | |  | Multivariate analysis | |
| --- | --- | --- | --- | --- | --- | --- |
|  |  | Hazard ratio (95% CI) | P value |  | Hazard ratio (95% CI) | P value |
| ACSL3 | 294 | 1.487 (1.069-2.069) | **0.019** |  | 1.306 (0.865-1.970) | 0.204 |
| BIRC5 | 294 | 1.252 (1.049-1.493) | **0.013** |  | 1.384 (1.053-1.819) | **0.020** |
| FTH1 | 294 | 1.522 (1.123-2.065) | **0.007** |  | 1.305 (0.925-1.843) | 0.130 |
| HIF1A | 294 | 1.303 (1.076-1.578) | **0.007** |  | 1.296 (0.997-1.684) | 0.053 |
| TOP2A | 294 | 1.152 (0.988-1.342) | 0.070 |  | 0.782 (0.589-1.038) | 0.089 |
| VEGFA | 294 | 1.324 (1.031-1.700) | **0.028** |  | 1.074 (0.752-1.535) | 0.695 |
